# Origin, behaviour, and genetics of reproductive workers in an invasive ant

**DOI:** 10.1186/s12983-021-00392-2

**Published:** 2021-03-22

**Authors:** Pauline Lenancker, Heike Feldhaar, Anja Holzinger, Melinda Greenfield, Angela Strain, Peter Yeeles, Benjamin D. Hoffmann, Wee Tek Tay, Lori Lach

**Affiliations:** 1 College of Science and Engineering, James Cook University, Cairns, QLD 4870 Australia; 2 CSIRO, Tropical Ecosystems Research Centre, Darwin, NT 0822 Australia; 3 Animal Ecology I, Bayreuth Centre of Ecology and Environmental Research, University of Bayreuth, 95440 Bayreuth, Germany; 4 CSIRO, Black Mountain Laboratories, Canberra, ACT 2601 Australia

**Keywords:** *Anoplolepis gracilipes*, Aggression tests, Eusocial insects, Haplodiploid, Hymenoptera, *Oecophylla smaragdina*, Oocytes, Ovaries, Worker conflict, Worker reproduction

## Abstract

**Background:**

Worker reproduction has an important influence on the social cohesion and efficiency of social insect colonies, but its role in the success of invasive ants has been neglected. We used observations of 233 captive colonies, laboratory experiments, and genetic analyses to investigate the conditions for worker reproduction in the invasive *Anoplolepis gracilipes* (yellow crazy ant) and its potential cost on interspecific defence. We determined the prevalence of worker production of males and whether it is triggered by queen absence; whether physogastric workers with enlarged abdomens are more likely to be reproductive, how normal workers and physogastric workers compare in their contributions to foraging and defence; and whether worker-produced males and males that could have been queen- or worker-produced differ in their size and heterozygosity.

**Results:**

Sixty-six of our 233 captive colonies produced males, and in 25 of these, some males could only have been produced by workers. Colonies with more workers were more likely to produce males, especially for queenless colonies. The average number of days between the first appearance of eggs and adult males in our colonies was 54.1 ± 10.2 (mean ± SD, *n* = 20). In our laboratory experiment, queen removal triggered an increase in the proportion of physogastric workers. Physogastric workers were more likely to have yolky oocytes (37–54.9%) than normal workers (2–25.6%), which is an indicator of fertile or trophic egg production. Physogastric workers were less aggressive during interspecific aggression tests and foraged less than normal workers. The head width and wing length of worker-produced males were on average 4.0 and 4.3% greater respectively than those of males of undetermined source. Our microsatellite DNA analyses indicate that 5.5% of worker-produced males and 14.3% of males of undetermined source were heterozygous, which suggests the presence of diploid males and/or genetic mosaics in *A. gracilipes*.

**Conclusions:**

Our experimental work provides crucial information on worker reproduction in *A. gracilipes* and its potential cost to colony defence. The ability of *A. gracilipes* workers to produce males in the absence of queens may also contribute to its success as an invasive species if intranidal mating can take place between virgin queens and worker-produced males.

**Supplementary Information:**

The online version contains supplementary material available at 10.1186/s12983-021-00392-2.

## Background

In social Hymenoptera, a caste of less reproductive individuals (i.e. workers) contributes to colony labour while fecund individuals (i.e. queens) produce offspring. However, workers sometimes challenge the reproductive primacy of the queen by producing male-destined eggs [1, 2]. In most species, including *Apis* honeybees, Meliponinae stingless bees, Vespinae wasps, *Bombus* bumblebees, and most ants, workers possess ovaries but cannot mate [2]. Through the haplodiploid sex determination system of Hymenoptera, in which females (i.e. workers and queens) are diploid and originate from fertilized eggs while males are haploid and originate from unfertilised eggs (arrhenotoky), these workers can produce male-destined eggs [3, 4].

Despite worker reproduction not being beneficial to social Hymenoptera queens, workers from queenright colonies produce males in 69 out of 90 taxonomically diverse studied species (ants, sweat bees, bumblebees, honeybees, stingless bees, and wasps) for which workers have functional ovaries [5]. Workers from most ant species have retained functional ovaries and are able to lay male-destined eggs [2, 6]. According to kin selection theory, worker reproduction is beneficial at the worker level because workers tend to be more related to their own sons (average degree of relatedness, r = 0.5) than to their brothers (i.e. queen’s sons, r = 0.25) [7]. However, in the case of queens mating multiple times, workers tend to be more related to their brothers (r = 0.25) than to other workers’ sons (r < 0.25) which favours workers to police eggs laid by other workers [8, 9]. The queen should always prefer to invest in her own sons, which are more related to her (r = 0.5) than her grandsons (r = 0.25). Queens from several ant species can inhibit worker fertility via pheromones and therefore, workers are most reproductive in the absence of a queen ([2] e.g. *Neoponera apicalis* [10]; *Camponotus floridanus* [11]; *Lasius niger* [12]). If the queen dies, the production of males by workers advantages both workers and queens because it is the last opportunity for the deceased queen to contribute to the gene pool.

Ant workers with functional ovaries can also produce trophic eggs (unviable eggs fed to the colony) [2, 13]. Trophic eggs are used to transfer proteins and nutrients to members of the colony (especially queens and larvae) and can be an important source of nutrition for colony members [10, 13–16]. In some species, workers switch from trophic egg to male-destined egg production in the absence of queens [2]. For example, *Oecophylla longinoda* workers produce trophic eggs in queenright colonies and begin laying male-destined eggs one to two months after being separated from the queen [17]*.*

The production of males by workers can be costly and disrupt the social organisation of the colony [18]. Colony productivity may decrease due to workers laying male-destined eggs and exhibiting high levels of aggression toward other workers instead of contributing to colony labour [18–20]. For example, worker reproduction led to a 15% reduction in time spent on brood care for queenless colonies of *Temnothorax allardycei*, while worker dominance behaviour to regulate worker reproduction in queenless colonies of *Pachycondyla obscuricornis* incurred an energetic cost and a reduction in colony labour [19, 20]. In the case of non-invasive ants, worker reproduction is often associated with queen death or colony decline [2].

Given the presumed costs of worker reproduction, we would not expect invasive ants to have reproductive workers. However, worker reproduction was recently reported for the first time in the yellow crazy ant (*Anoplolepis gracilipes*) [21], one of the world’s worst invaders and for which the reproductive mode is not fully resolved [21–23]. Workers with an unusually distended abdomen (i.e. physogastric), from queenless *A. gracilipes* colonies collected in Taiwan, had ovaries that were more developed than those of other workers and laid male and trophic eggs [21]. Worker-produced males may produce viable sperm, but we do not know their relative fitness compared to queen-produced males. Ploidy and male size can be indicators of fitness as diploid males tend to be sterile [3, 4, 24] and male size is correlated with fitness in some ant species [25, 26]. Results from several genetic studies suggest that heterozygous males are common in this species across its range (Borneo [23], Christmas Island [27], Arnhem Land in Australia [28, 29], Taiwan [21]) which would suggest *A. gracilipes* males are often diploid.

We do not know whether worker reproduction contributes to or hinders the invasive success of *A. gracilipes*. Worker reproduction in this highly successful invader [22, 30] may be too rare to impose a cost on colony success, or the benefits of worker reproduction (e.g. production of fertile males) may outweigh its costs (e.g. reduction in colony labour). Understanding colony dynamics when the queen dies and/or the colony declines may provide insights relevant to the management and control of this invasive species.

We used a combination of observations, experiments, microscopy, and genotyping to investigate the attributes, potential triggers, and costs of worker reproduction in *A. gracilipes*. Our specific aims were to determine 1) how common worker production of males is in *A. gracilipes* colonies and whether it is triggered by queen absence; 2) whether physogastric workers are more likely to be reproductive; 3) how physogastric and normal workers compare in their contributions to foraging and defence; and 4) whether worker-produced and queen-produced males differ in their size.

## Results

### Dissections of worker’s ovaries of entire colonies

Workers with two to four exposed intersegmental membranes (hereafter physogastric workers) had a conspicuously enlarged gaster that was more likely to contain yolky oocytes (54.9%, *N* = 56/102), which may indicate the presence of fertile or trophic eggs [13, 31], than workers that had zero to one exposed intersegmental membrane (hereafter normal workers, 25.6%, *N* = 10/39, GLM: binomial, ANOVA: χ2 = 4.7578, df = 1, *p* = 0.0292). The proportion of physogastric workers was higher in queenless (mean ± SD: 78.6 ± 10.4%) than in queenright colonies (mean ± SD: 53.9 ± 11.6%, GLM: binomial, ANOVA: χ2 = 14.093, df = 1, *p* = 0.0002). Yellow bodies, which are characteristic of fertile eggs but are sometimes observed in trophic egg-layers [13, 32–34], were only observed in physogastric workers (10.7% in queenright colonies and 12.9% in queenless colonies).

### Colony observations

**Fig. 1:**
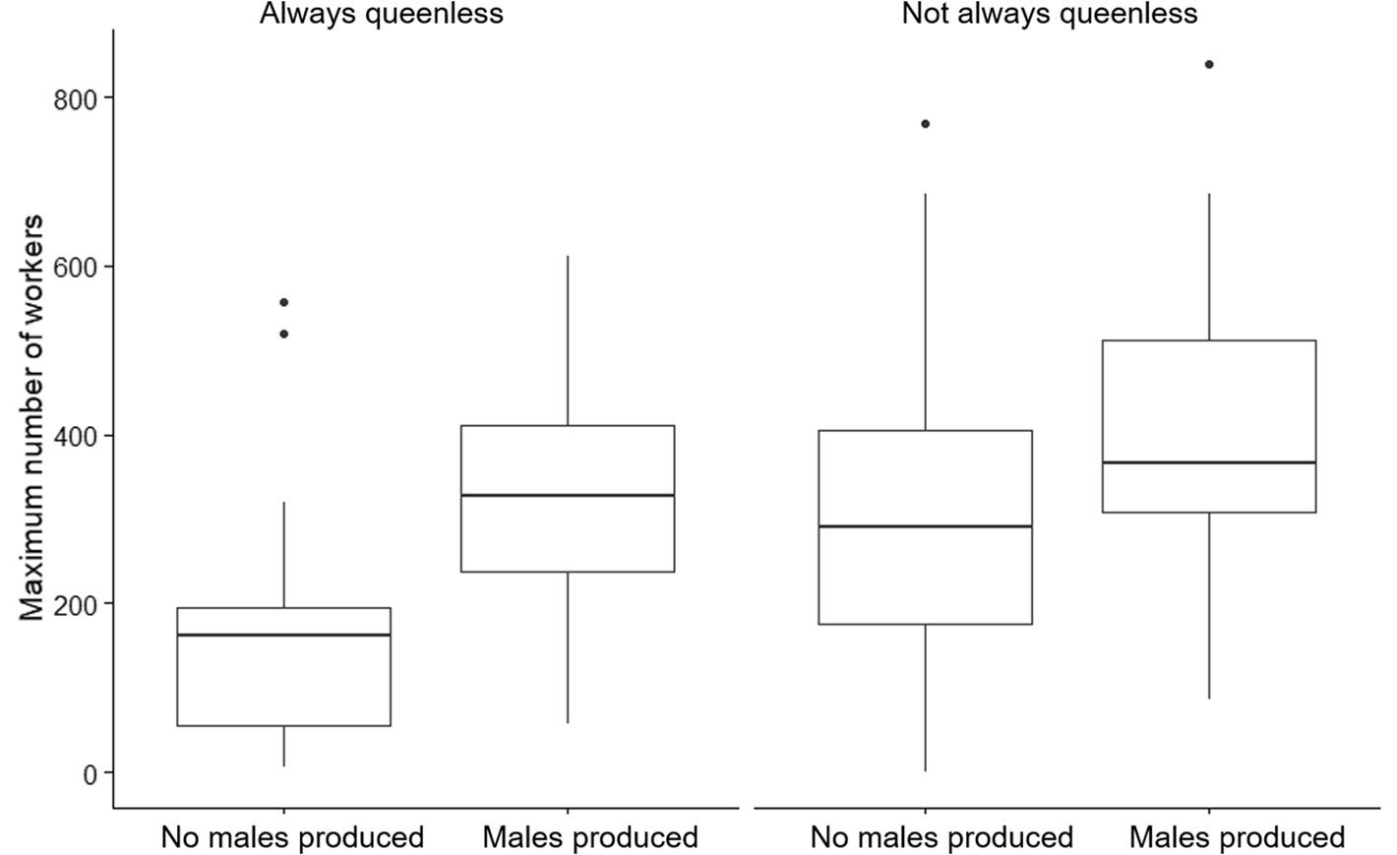
Male production in the 233 observed laboratory colonies by whether colonies were queenless and the maximum number of workers in the colony during captivity. See Table 1 for statistical results

We observed males in 66 out of our 233 captive colonies (28%). Of the 66 colonies in which males were observed, 44 were queenless throughout their captivity, and 22 had at least one queen at some time in their captivity. Of the 22, 14 had at least one queen present when males were first observed. Of the 167 colonies that never produced males, 70 were always queenless. Colonies with more workers were more likely to produce males, especially if these colonies were queenless. Male production was not significantly predicted by time in captivity regardless of whether a colony was ever queenright (Fig. 1, Table 1).

**Table 1:** Summary of generalized linear mixed model (GLMM), generalized linear model (GLM) or cumulative link mixed models (CLMM) results for each response variable for analyses of 1) colony observations, 2) the queen transfer experiment, and 3) aggression tests. ‘x’ represents the interaction term  * *p* < 0.05, ** *p* < 0.01, *** p<0.001

Response and explanatory variables	df	χ2 or LRstat	***p***
**1. Male production, GLMM binomial,** ***n*** **= 233 colonies**			
Whether the colony ever had a queen	1	2.28	0.13
Maximum number of workers	1	35.35	< 0.0001***
Days in captivity	1	3.17	0.08
Whether the colony ever had a queen x max. Number of workers	1	7.58	< 0.0059**
Whether the colony ever had a queen x days in captivity	1	3.04	0.08
**2. Proportion of normal workers until day 60, GLMM binomial,** ***n*** **= 100 observations and 10 colonies**			
Colony status (queenright or queenless)	1	1.80	0.18
Time (since the beginning of the experiment)	4	15.57	0.0037**
Colony status x Time	4	14.49	0.0059**
**Proportion of normal workers until day 120, GLMM binomial,** ***n*** **= 54 observations and 3 colonies**			
Colony status (queenright or queenless)	1	0.0001	0.99
Time (since the beginning of the experiment)	8	47.26	< 0.0001***
Colony status x Time	8	37.92	< 0.0001***
**3. Maximal aggression score, CLMM,** ***n*** **= 70**			
Colony status	1	5.66	0.0173*
Worker type	1	6.65	0.0099**
Colony status x Worker type	1	1.30	0.25
**Survival of all three** ***A. gracilipes*** **workers, GLMM negative binomial,** ***n*** **= 70**			
Colony status	1	2.45	0.12
Worker type	1	0.33	0.57
Colony status x Worker type	1	1.83	0.18
**Survival of the** ***O. smaragdina*** **worker, GLMM binomial,** ***n*** **= 70**			
Colony status	1	14.45	< 0.0001***
Worker type	1	1.53	0.22
Colony status x Worker type	1	6.19	< 0.0129*
**Species initiating the fight, GLM binomial,** ***n*** **= 51**			
Colony status	1	4.12	0.0423*
Worker type	1	5.25	0.0220*
Colony status x Worker type	1	0	0.99
**Presence absence of mature oocytes, GLMM binomial,** ***n*** **= 210**			
Colony status	1	5.52	0.01876*
Worker type	1	20.40	< 0.0001***

In 25 of the 66 colonies with males produced, we could attribute at least some of the adult males to workers. All 25 of these colonies came into captivity without queens, so we could not determine how long they were queenless before males were produced, but we could discern that the brood that produced males later in captivity were from workers. Seven of these 25 colonies also had males emerge earlier in captivity from brood with which they entered captivity and for which we therefore could not rule out a queen origin. We also could not rule out a queen origin of males for an additional 36 colonies that either came in queenless (22 colonies) or became queenless during captivity (1 colony) or were never queenless (14 colonies). In five of the 66 colonies, the males could have been produced by workers or alate queens.

We could attribute eggs, larvae, or pupae to workers in 35 colonies. On 20 occasions (in 19 colonies), we could trace the maturation of worker-produced brood from egg to adult male. The number of days from the first sighting of worker-produced eggs to the first sighting of adult males was 54.1 ± 10.2 days (mean ± SD). The number of workers in the colony on the day that worker-produced eggs were first observed was estimated as 10–541 (median = 180, *n* = 31).

We observed eight colonies in which one or more alate queens co-occurred with males. In six of these the queens emerged in captivity so we knew there was no prior exposure to males. In five of the six colonies in which the queens emerged in captivity, the queens subsequently lost their wings (a possible indicator of mating) and in all of these, eggs appeared over subsequent weeks. We could not determine whether these were laid by workers or queens.

### Male morphometry and genotyping

**Fig. 2:**
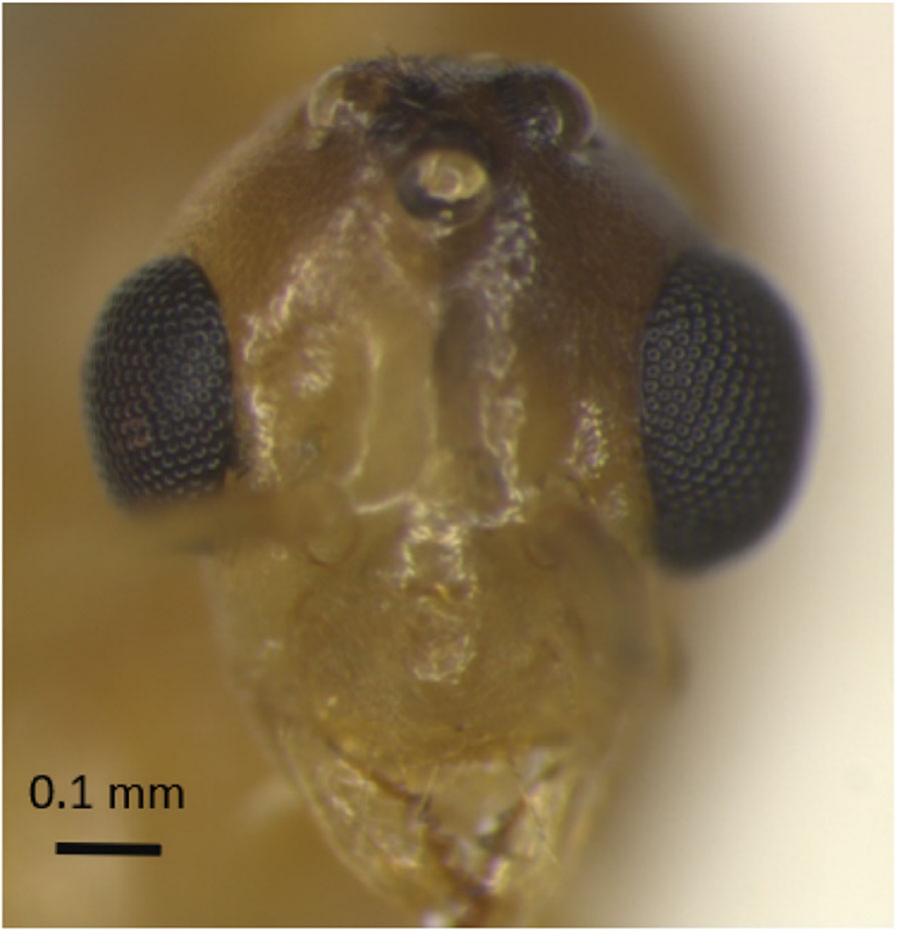
Head of a male *A. gracilipes* with a normal eye on the left and an oversized eye on the right

Head width and wing length, but not Weber’s length, or wing width, of worker-produced males were significantly greater than for males of undetermined source (i.e. males that could have been produced by dealate queens or alate queens or workers, Table 2). The Weber’s length of worker-produced males tended to be greater than for males of undetermined source (Table 2). Six worker-produced males had conspicuous deformities: 2 out of 55 worker-produced males for which we measured the head width had one eye that was oversized compared to the other eye (Fig. 2), and 4 out of 58 worker-produced males with intact wings had abnormal wings (black and stubby wings or underdeveloped wing tips). We successfully genotyped four of these deformed males and they were all hemizygous (Additional file 1: Table S1). Two out of 17 males of undetermined origin for which we measured the head had one oversized eye (Fig. 2) but none were genotyped successfully (Additional file 1: Table S1). We did not observe other conspicuous morphological anomalies on males with an oversized eye.

**Table 2:** Trait measurements in mm of worker-produced and males of undetermined source^a^ (mean ± SD) and results from type II Wald tests on LMM (df = 1) * *p* < 0.05, ** *p* < 0.01

Trait	Worker-produced		Undetermined source^a^		χ2	***P***
Head width	0.77 ± 0.04	*n* = 55	0.74 ± 0.03	*n* = 24	10.423	0.0012**
**Weber’s length**	1.79 ± 0.12	*n* = 83	1.72 ± 0.12	*n* = 30	3.7443	0.053
**Wing width**	1.37 ± 0.10	*n* = 50	1.30 ± 0.10	n = 30	0.2135	0.644
**Wing length**	3.11 ± 0.20	n = 54	2.98 ± 0.22	n = 21	4.3492	0.037*

^a^ Males that could have been produced by queen (dealate or alate) or workers

Most worker-produced males and males of undetermined source were hemizygous and more than one worker produced males in at least one of the queenless colonies. We found that 5.5% of worker-produced males (*n* = 3/55 males from 2/12 colonies), 14.3% of males that could have been produced by dealate queens or workers (*n* = 2/14 males from 2 colonies out of 3), and 0% of males that could have been produced by alate queens or workers (*n* = 0/6 from 2 colonies) were heterozygous for at least one locus (Additional file 1: Table S1). In 9 colonies we genotyped more than one worker-produced male and found three different alleles at Ano5 in one of these colonies (Additional file 1: Table S1), which indicates that more than one worker produced males in this colony.

All workers were heterozygous for at least one locus while most queens were homozygous. We found that all (*n* = 48/48 workers from 13/13 colonies) the genotyped workers from colonies in which males were worker-produced were heterozygous, as were all workers (*n* = 16/16 workers from 4/4 colonies) from colonies in which males could have been produced by dealate queens or workers, and all workers (*n* = 10/10 workers from 2/2 colonies) from colonies in which males could have been produced by alate queens or workers (Additional file 1: Table S1). Eight queens out of nine were homozygous at all loci and one queen was heterozygous at Ano4 (Additional file 1: Table S1). Note that amplification failures (Additional file 1: Table S1) were due to some individuals being already dead and potentially degraded at the time of collection.

### Queen transfer experiment

**Fig. 3:**
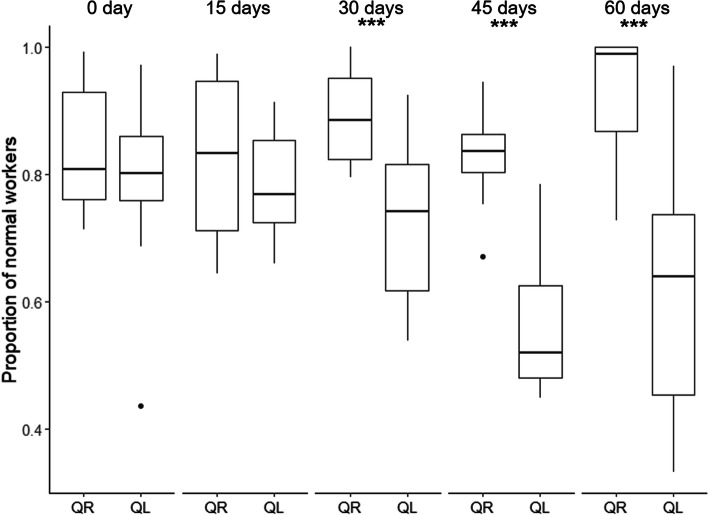
Proportion of normal workers in the queen transfer experiment by colony status (QR = queenright, QL = queenless, N = 10 for each) and number of days since the start of the experiment until day 60. *** indicates a significant difference between queenright and queenless colonies for the corresponding time (GLMM: binomial, Table 1, post-hoc tests ***p < 0.001)

The number of physogastric workers increased following queen removal (Table 1, Fig. 3, Additional file 2: Fig. S1). We found similar proportions of physogastric workers in queenright and queenless subcolonies from days 0 and 15 of the experiment (Fig. 3, Table 1, post hoc tests *p* = 0.1803 for day 0 and 0.1669 for day 15). At days 30, 45, and 60, there were more physogastric workers in queenless than in queenright colonies (Fig. 3, Table 1, post hoc tests *p* = 0.0005 for day 30, *p* = 0.0001 for day 45, and *p* < 0.0001 for day 60).

Workers in the queenless subcolonies readily accepted the return of their corresponding queen at day 60. The queens were either ignored or touched by workers for the first hour after translocation (aggression score 0–1, [35]), and all six queens were tended by workers (aggression score 1) inside a nest or under the egg carton after 24 h. Three queens survived until day 120.

The number of physogastric workers increased in the newly queenless subcolonies (hereafter referred to as secondarily queenless) following queen transfer. Initially, secondarily queenless subcolonies had significantly fewer physogastric workers compared to secondarily queenright subcolonies (day 75 *p* = 0.0071, day 90 p = 0.0001, Additional file 2: Fig. S1). The trend reversed from day 105 and physogastric workers became significantly more common in the secondarily queenless subcolonies at day 120 (day 105 *p* = 0.406, day 120 *p* = 0.0481), although there was large variation, probably resulting from the small number of surviving colonies (*n* = 3, Additional file 2: Fig. S1). At day 120, secondarily queenless subcolonies had 53 to 467 workers per colony and secondarily queenright subcolonies 42 to 113. Yolky oocytes were not significantly more common in physogastric workers (mean ± SD: 41.7 ± 30.5%, *n* = 119) than in normal workers (mean ± SD: 24.1 ± 25.9%, *n* = 52) dissected at 120 days (GLMM: binomial, Type II Wald test: χ2 = 3.5061, df = 1, *p* = 0.0611). Only the ovaries of physogastric workers had yellow bodies (7.8% had yellow bodies in queenright and 8.6% in queenless colonies).

We observed a difference in behaviour between normal and physogastric workers. During colony monitoring, we observed normal workers in the foraging area outside of the nesting tubes more often than physogastric workers (in 81/100 observations of normal workers and 5/100 of physogastric workers) regardless of whether the colony was queenright or queenless (GLMM: binomial, Type II Wald test, worker type: χ2 = 55.5766, df = 1, *p* < 0.0001, colony status: χ2 = 0.6344, df = 1, *p* = 0.4257). Queens continued to produce brood throughout the experiment. We did not observe trophic eggs in the presence or absence of a queen in any of the colonies. We did not observe male production in any of the colonies, including the six queenless colonies we monitored for the additional 60 days (i.e. until day 180). These six colonies had 40 to 337 workers at the end of the observations (mean ± SD = 142.3 ± 91.9) and 36 to 305 of these workers were physogastric (118 ± 87.72).

### Aggression tests

**Fig. 4:**
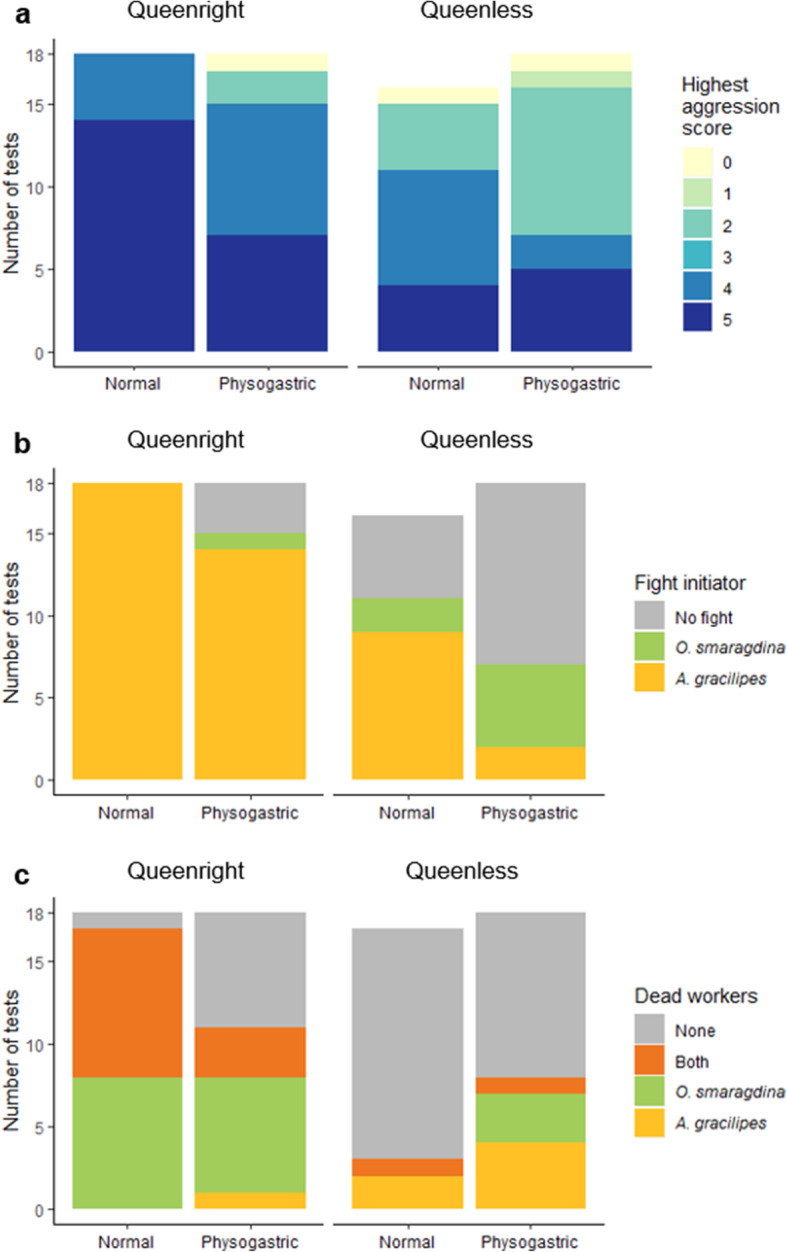
Results from the aggression tests between *O. smaragdina* workers and *A. gracilipes* normal or physogastric workers from queenright or queenless colonies. a: Highest aggression score for each trial, b: Number of fights initiated by *A. gracilipes* and *O. smaragdina* workers, c: Number of fights that resulted in the death of the *O. smaragdina* worker and/or the death of one of the three *A. gracilipes* workers. See Table 1 for statistical results

Physogastric workers and workers from queenless colonies irrespective of whether they were physogastric or normal were less aggressive than normal workers and workers from queenright colonies. The highest aggression scores of trials with normal workers (Fig. 4a, median: 5, range: 0–5, *N* = 34) was higher than the aggression score of trials with physogastric workers (median: 4, range: 0–5, *N* = 36, Table 1). The highest aggression score of trials with workers from queenright colonies was also higher (Fig. 4a, median: 5, range: 0–5, N = 36) than when the workers were from queenless colonies (median: 4, range: 0–5, N = 34, Table 1). The interaction between worker type and colony status was not significant (Table 1). *Anoplolepis gracilipes* workers were more likely to initiate the fight in interspecific aggression tests against *Oecophylla smaragdina* if they were from queenright colonies as opposed to queenless or were normal workers as opposed to physogastric (Fig. 4b, Table 1). *Oecophylla smaragdina* workers were less likely to survive if they were fighting against normal workers vs. physogastric workers from queenright colonies, but physogastry did not affect their survival if *A. gracilipes* workers originated from a queenless colony (post hoc tests *p* < 0.05 between normal and physogastric workers from queenright colonies and *p* > 0.05 from queenless colonies) (Fig. 4c, Table 1). The survival of *A. gracilipes* was not influenced by physogastry or whether they originated from a queenless or queenright colony (Fig. 4c, Table 1). Physogastric workers dissected after the aggression trials were more likely to have yolky oocytes (*N* = 40/108) than normal workers (*N* = 2/102, Table 1). We only observed yellow bodies in physogastric workers (9.3% had yellow bodies in queenright colonies and 12.3% in queenless colonies).

## Discussion

Our experimental work significantly improves our understanding of worker reproduction in the invasive *A. gracilipes*. We could attribute male production to workers in 25 captive colonies out of 66 male-producing colonies. In our queen transfer experiment, the absence of a queen triggered an increase in physogastric workers suggesting the existence of queen control over worker reproduction. Dissections of physogastric workers revealed that their ovaries were more likely to contain yolky oocytes. We also found that physogastric workers were less aggressive and less likely to forage than normal workers, which indicates that their presence may be costly to colony foraging capacity and defence. The head width and wing length of worker-produced males were slightly larger than males for which we could not rule out a queen or worker origin. Finally, 5.5% of worker-produced males and 14.3% males that could have been produced by dealate queens or workers were heterozygous. We found that most queens were homozygous while most workers were heterozygous for at least one locus. This indicates that the reproduction of *A. gracilipes* is unusual and may involve diploid males and/or gynandromorphs, consistent with previous suggestions [23, 29]. To the best of our knowledge, this study is the first to test for the potential cost of worker reproduction in an invasive ant species. Given the cost of worker reproduction, male-production by workers is unexpected in a highly successful invasive species such as *A. gracilipes*, which may indicate that worker reproduction has benefits.

### Frequency of worker reproduction and evidence of queen control

Male production by workers occurred in 25 colonies but not in another 70 colonies that were always queenless. Nor did we find male production by workers within any of the colonies from the queen transfer experiment, including six queenless colonies that we monitored past the end of the experiment and that had been queenless for 116 to 186 days. The absence of male production in these colonies could be due to the low worker count, as we found from our observations of 233 captive colonies that worker number was positively associated with male production. In Taiwan, three queenless colony fragments out of nine produced male brood after being kept for four months in the laboratory, and adult males were observed in one of these fragments two months later [21]. This matches our observation that it took 54.1 ± 10.2 days (mean ± SD) for males to develop from eggs to adult. Our observations on male production are all based on laboratory-kept colonies. Caution must be taken when extrapolating to field colonies, which are not as likely to be queenless*,* although queenless aggregations of *A. gracilipes* workers and brood are frequently observed in the field (personal observation).

Additional results from our colony observations suggest that queens may limit worker reproduction, though we cannot rule out worker policing. In our queen transfer experiment, removing queens triggered an increase in physogastric workers, and moving the queen back after 60 days led to a decrease in physogastric workers. Observations from Lee et al. (2017) [21] suggest that physogastric workers may switch from producing trophic eggs in queenright conditions to producing viable male eggs in queenless conditions. Workers of several ant species switch from trophic egg production to male egg production when the queen dies or disappears (e.g. *Aphaenogaster senilis* [36]; *Aphaenogaster cockerelli* [37]; *Prolasius advena* [38]; *N. apicalis* [10]; *O. longinoda* and *O. smaragdina* [17]*,* but to the best of our knowledge, *A. gracilipes* is the only invasive ant species that has been found to do so. Social insect queens can inhibit worker reproduction with queen pheromones, i.e. chemical signals indicating the reproductive status of the queen [2, 39]. Several experiments with ants, wasps, and some bees have shown that applying synthetic queen pheromones to queenless colonies inhibits worker reproduction by preventing workers from activating their ovaries and by causing secondary oocyte resorption [12, 39–41]. Worker reproduction could also be controlled through the policing of reproductive workers [5, 8]. For example, queens and workers could behave aggressively towards egg layers or destroy worker-laid eggs [9].

Physogastric workers dissected as part of the dissections of entire colonies and following the aggression tests were more likely to have yolky oocytes than normal workers. Yolky oocytes indicate the presence of fertile or trophic eggs [13, 31]. There was no difference in yolky oocyte presence at the end of the queen transfer experiment, probably because colonies in the queen transfer experiment had been queenless for a relatively short period (60–120 days vs 108 and 143 days for dissections of worker ovaries of entire colonies and 102–212 days for aggression tests). We only observed yellow bodies in physogastric workers (7.8–12.9%). Yellow bodies can indicate active oviposition of viable eggs, although they are sometimes observed in trophic egg-layers [13, 32–34]. Physogastric *A. gracilipes* workers originating from Taiwan also had a higher reproductive potential than normal workers [21]. They had more well-developed ovaries and more yolky oocytes than normal workers [21]. Yellow bodies were also only observed in physogastric workers (13%) [21]. Histological sections of the abdomen of physogastric workers indicated that fat bodies were more abundant in physogastric than in normal workers [21]. The distended abdomen of physogastric workers could therefore be due to the presence of fat bodies and well-developed ovaries. The reproductive or trophic egg-layer status of individual workers can only be determined by ovary dissections or by observations of egg-laying. Physogastric workers are more likely to be reproductive than normal workers, but some non-reproductive workers may have a temporarily distended abdomen from feeding extensively on liquids. Additional research is needed to determine an objective way to non-destructively distinguish reproductive from non-reproductive workers.

### Costs of worker reproduction

We found that behavioural differences between physogastric and normal workers may decrease the competitive ability of the colony. Physogastric workers in queenright and queenless colonies were infrequently observed in the foraging area and were mostly observed inside the nesting tubes during the queen transfer experiment. These observations suggest that physogastric workers do not contribute to foraging as much as normal workers and may spend more time tending to the brood than contributing to foraging activities. Physogastry may also affect the ability of workers to defend the colony during interspecific conflicts. We found that physogastric workers were less aggressive towards *O. smaragdina* workers and were less likely to engage in a fight than normal workers, which would reduce the potential of *A. gracilipes* colonies with a large proportion of physogastric workers (such as queenless colony fragments) to become behaviourally dominant. In queenright colonies, the queen may limit the proportion of physogastric workers and thus minimize the costs associated with worker reproduction, such as a decrease in foraging and defence activities.

The production of males by workers also generates costs for other ant species [18]. For example, in *Neoponera obscuricornis* colonies, two costs are associated with worker reproduction following queen removal: an increase in energetic cost associated with aggressive interactions between workers for egg-laying and a decrease in colony labour due to reproductive workers spending less time working for the colony [19]. Costly worker conflicts about which workers become reproductive and which workers continue to contribute to colony labour also take place in *Aphaenogaster senilis* [36]. We have never observed aggressive interactions among workers so it is unlikely conflicts take place among *A. gracilipes* workers as to which will become physogastric and which physogastric workers will produce males. Adult males do not appear to originate from a single dominant physogastric worker in queenless *A. gracilipes* colonies, as our genetic results indicated that males originated from more than one worker in at least one of our queenless colonies. This result is consistent with male genetic data for *A. gracilipes* in Taiwan, which showed four different alleles at one locus (Ano10) in one queenless colony fragment [21].

Without a queen, *A. gracilipes* colonies are doomed because reproductive workers are unable to lay worker eggs due to their lack of spermatheca [21]. The only chance of survival for a queenless colony would be to merge with a queenright colony and/or adopt a queen. Our workers in queenless colonies readily accepted their original queen back in the nest after being separated for 60 days. In the Northern Territory (Australia), laboratory-kept *A. gracilipes* queenless colonies were successfully merged with queenright colonies from a different source colony [42]. Orphaned colonies may therefore merge with other colonies and/or adopt a queen from a different colony in the field. However, the increase in proportion of physogastric workers, which have less competitive ability and do not contribute to foraging as much as normal workers, following queen death could precipitate the demise of orphaned colonies before such opportunity arises.

### Potential benefits of worker reproduction

Despite the costs associated with their lack of contribution to foraging and defence, the role of physogastric workers as trophic-egg layers in queenright colonies may be significant [21]. Colony observations have shown that trophic eggs may represent a major part of the larval diet in *A. gracilipes* [21]. We did not observe trophic eggs during the queen transfer experiment, but any trophic eggs produced by physogastric workers would likely have been fed to the queen and brood immediately after being laid, as observed in queenright *A. gracilipes* colonies by Lee et al. (2017) [21].

Worker reproduction may also increase the fitness of deceased *A. gracilipes* queens and orphaned workers because it is their last opportunity to contribute to the gene pool. In Taiwan, the seminal vesicles of *A. gracilipes* worker-produced males contained viable sperm suggesting that they are able to mate [21]. Although the reproductive mode of *A. gracilipes* is unresolved, genetic data and laboratory observations suggest that intranidal mating is the main mode of reproduction for this species [27, 43]. In eight of our captive colonies in which males were present, we observed alate queens with no prior exposure to males lose their wings before observing eggs in the colony. If queen brood or virgin queens were present in the colony at the time of the queen’s death and did not inhibit the production of males by workers, intranidal mating between worker-produced males and virgin queens could occur. Such a strategy could prolong the life of a colony after the queen’s death.

### Size and genotypes of males and implications for *A. gracilipes* reproduction

The head width and wing length of worker-produced males were significantly larger (4.0–4.3%) than for queen or worker-produced males and Weber’s length tended to be larger, which may provide worker-produced males with a competitive advantage [26]. We do not know whether *A. gracilipes* queens select the males they mate with, whether this selection involves male sizes, and whether larger males have a competitive advantage over smaller ones. Larger males of some *Pogonomyrmex* harvester ants are more successful at mating than smaller males because they can be more successful at gaining access to a mate and transfer a greater proportion of their sperm [25, 26]. It would be informative to test whether *A. gracilipes* queens choose larger males, potentially selecting worker-produced over queen-produced males.

We also found that 4.9% of worker-produced males and 21.1% of males that could have been produced by dealate queens or workers were heterozygous. Our findings are different to those of Lee et al. (2017) [21] who found all 14 *A. gracilipes* worker-produced males from a single queenless colony fragment to be hemizygous, and most of the 20 males from one queenright colony to be heterozygous. Elsewhere they have been genotyped, field-collected heterozygous *A. gracilipes* males were found to be common (Borneo [23], Christmas Island [27], Arnhem land Australia [29]). For example, about 50% of males collected in Borneo were heterozygous [23]. A heterozygous genotype in males would typically indicate diploidy.

In ant populations, when a queen mates with a male sharing the same genotype at the sex determination locus (or loci, i.e. match mating), half of the diploid offspring produced by the queen will be homozygous at the sex determination locus (or loci) and develop into diploid males instead of workers [4, 24]. Diploid male production is especially common in ant populations that have low genetic diversity (such as invasive populations), and hence low sex determining allele diversity [4, 24]. Intranidal mating may be common in *A. gracilipes* [27, 43] which would increase the chance of mating between related individuals and increase diploid male production.

Heterozygous *A. gracilipes* males that were produced by queens can be diploid, but it is unlikely that heterozygous males produced by workers are diploid. *Anoplolepis gracilipes* workers do not possess a spermatheca and are unable to mate [21] so their male offspring cannot be diploid through match mating. Instead, heterozygous males may be produced as a result of genetic mosaicism in which an individual possesses two distinct genotypes i.e. two sets of cells that are genetically different and spread across the body [44, 45]. In the case of *A. gracilipes* worker-produced males, heterozygous individuals could be hemizygous but combine the two genomes of a single worker, which would explain why they possess two different alleles at some loci.

Some heterozygous males produced by queens may also not be diploid but genetic mosaics. Diploid males tend to be sterile [3, 4, 24], but in some ant species, a low proportion of diploid males produce sperm and can father triploid progeny [46, 47]. In *A. gracilipes*, dissections of the seminal vesicles of 16 putative diploid males revealed that all of them possessed motile sperm, which suggests that they are not sterile [21]. Given that heterozygous males (putatively diploid) are apparently common for this species [this study, 23,27,29], we would expect a high prevalence of triploid workers resulting from successful mating between a queen and a diploid male. However, evidence of triploid *A. gracilipes* individuals has never been reported [21, 23, 27, 29, 48]. Heterozygous males may therefore be genetic mosaics with both maternal and paternal cells (i.e. gynandromorphs) [29]. Gynandromorphs, can occur in Hymenoptera and may combine the morphological features of males and females [49–51]. Some gynandromorphs can have bilateral symmetry with one side female and the other male, while other gynandromorphs are mosaics with male and female tissues spread across the body [45, 52]. The four males which had one eye that was oversized compared to the other eye (Fig. 2) may be sex mosaics with a conspicuous phenotype. In ants, sex mosaics sometimes present an enlarged eye (female) on one side of the head and a smaller eye (male) on the other side [50, 52].

In addition to gynandromorphs, *A. gracilipes* reproduction may also involve a caste determination system. We found that most genotyped workers were heterozygous for at least one locus, and that most queens were homozygous. This genetic pattern is typical of *A. gracilipes* populations and suggests that female castes are determined by a genetic component for this species [21, 23, 27, 29, 48]. A potential caste determination system could be linked to gynandromorphy in males. Queen-produced gynandromorph males could produce sperm from their inherited paternal or maternal cells, and female castes could be determined by a combination of male and female alleles [29]. The reproductive mode of *A. gracilipes* may contribute to the ecological dominance of this ant by maintaining a high number of heterozygous workers that may be better adapted to human-modified environments, as has been suggested for another invasive ant species, *Wasmannia auropunctata* (the little fire ant or electric ant) [53, 54].

## Conclusions

We found that workers produced males in at least 25 of our 233 captive *A. gracilipes* colonies. Our work suggests that queen removal triggers workers to become physogastric and potentially reproductive. Physogastric workers can be costly to colony foraging capacity and defence because they forage less and are less aggressive in interspecific conflicts than normal workers. However, reproductive workers may also benefit the colony when the queen dies because worker-produced males are the last opportunity for workers and the deceased queen to contribute to the gene pool. Worker-produced males were slightly larger and less likely to be heterozygous than males that could have been produced by queens or workers. Our results are consistent with those of other studies that suggest that the reproductive mode of *A. gracilipes* is unusual and may involve gynandromorphy and/or a caste determination system [23, 29, 48]. Additional investigations into the reproductive mode of *A. gracilipes* are necessary to resolve current uncertainties on worker and queen caste determination as well as the occurrence of gynandromorphs and to determine whether this potentially unusual reproductive mode contributes to the invasive success of *A. gracilipes*.

## Methods

### Colony collection and worker dissections

Colonies of *A. gracilipes* used in all our experiments were collected in Queensland, Australia, which is part of the invasive range of this species (Additional file 1: Table S2). Nests were visually located and partially excavated to collect queens, workers and brood. For each worker’s dissections in our experiments, we counted the number of ovarioles and yolky (i.e. opaque) oocytes and determined the presence of yellow bodies. We avoided bias for all ovary dissections by keeping the dissector (PL in all cases) blind to colony status (queenless or queenright) and aggression test outcome (see aggression tests section) for the dissections following aggression tests.

### Dissections of worker’s ovaries of entire colonies

We dissected the ovaries of all the workers from two queenright colonies that had been captive for 175 and 349 days (26 and 29 workers per colony containing 6 and 8 queens, respectively, Additional file 1, Table S3). We also dissected all the workers (22 and 64 workers) from two colonies that had been captive for 125 and 368 days and queenless for 108 and 143 days respectively following the queens’ death (Additional file 1: Table S3). Our aim was to objectively define physogastry and determine whether there is a link between workers’ physogastry and their reproductive capability.

Colonies were reared in a 150x220x320mm (height x width x depth) box with two 50 ml nesting tubes (length x diameter: 93x60mm) containing moist cotton and a 35x100x150mm (height x width x depth) piece of cardboard egg carton. The colonies were kept in a constant temperature room at 26 ± 0.2 °C (mean ± SD) and 59.5 ± 4.4% humidity.

**Fig. 5:**
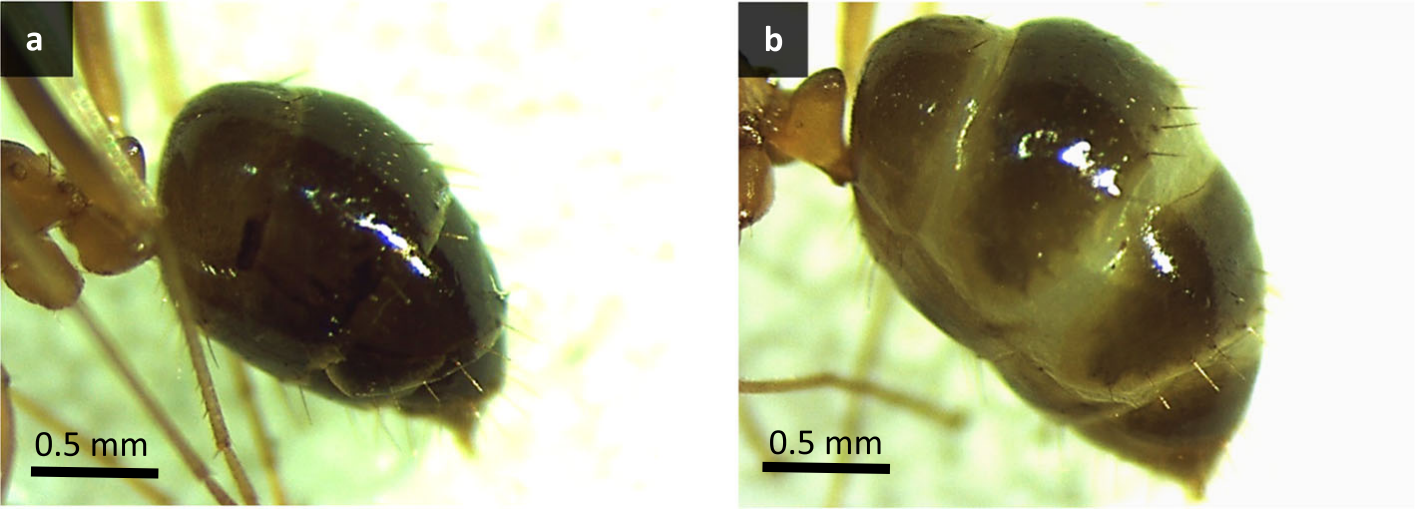
External morphology of the abdomen of (a) a normal worker and (b) a physogastric worker. Note the exposed intersegmental membranes of the physogastric worker

The colonies were alternately fed either a mealworm or a cricket twice a week and 25% sugar water ad-libitum. Prior to dissecting each worker, we visually evaluated its physogastry by counting the number of exposed intersegmental membranes on its gaster (Fig. 5). In accordance with our results (see Results section), we define physogastric workers as having a conspicuously enlarged gaster with two to four exposed intersegmental membranes. We used this definition of physogastric workers for the queen transfer experiment and the aggression tests.

### Colony observations

We conducted observations from May 2016 to December 2019 on 419 colonies collected from May 2016 to June 2019 at 23 sites in Queensland (20 near Cairns, 2 near Townsville, 1 near Hervey Bay, Additional file 1, Table S2 and S4). Most colonies were collected by placing transects of bamboo segments (approximately 300 mm long, 40-60 mm width of opening) at multiple sites and collecting pieces that *A. gracilipes* had colonized. Colonies were kept in the same conditions as described in the Dissections of worker’s ovaries of entire colonies section. We observed colonies every 1–3 weeks during which we counted the number of queens (alate and dealate) and males, categorized the number eggs, larvae, and pupae (0, 11–20, 21–50 and 51+). We did not distinguish between trophic eggs and reproductive eggs. To estimate the total number of workers during each observation, we categorized the number of workers (0–10, 11–20, 21–50, 51–100, 101–200 and 200+) engaged in each of five different activities (in the nest tube, in the sugar tube, walking around, standing still, other) and then summed the mid-points of the recorded categories across the five activities. To check for the presence of males, we scrutinized the nest and checked dead ant piles. We excluded from analyses colonies that had more than 21 days between any two observations, had fewer than 4 observations, had been in captivity for fewer than 26 days, or that were used for other experiments. The remaining 233 colonies had been observed 5–73 times (median = 32) for at least 29 and up to 747 days (median = 237) at intervals of 8–21 days (median = 10). The number of observations varies among colonies because it is dependent on their collection date and longevity.

For colonies that produced males, we categorized the likely male source based on the preceding observations of brood and queens. We concluded that any brood produced during a queenless (alate or dealate) state after at least three weeks in which no brood had been observed had been produced by workers. We could then usually trace the development from egg to larvae to pupae over successive weeks of observations and conclude that any males that emerged were worker-produced. Two colonies collected without queens had more disjunct patterns of observable eggs, but we concluded that their males were worker-produced considering they were in captivity queenless for 140 and 146 days and male brood does not enter diapause in constant laboratory conditions [55]. We approximated the time to development as the number of days from when we first observed a worker-produced egg to when we first observed an adult male on 20 occasions in 19 colonies.

Males that emerged in colonies in which queens had emerged, and in which brood appeared after at least three weeks in which no brood had been observed, were considered to have been produced by either workers or alate queens. Males that emerged in colonies (either queenless or queenright) in which brood was present continuously following collection, were considered to be either queen- or worker-produced. We collected some live males for genotyping, but otherwise left males in colonies so as to disrupt functioning as little as possible. Workers and queens were collected dead opportunistically to minimize effects on colony dynamics. None of the colonies were entirely genotyped.

### Morphometry and genotyping

#### Measurements

Live and dead adult males that were found during the colony observations (protocol described above) were collected, placed in ethanol, and measured under a Leica M165C stereomicroscope at 20-80x magnification. We measured the head width (i.e. maximum width of the head in full face view including the eyes), Weber’s length, wing width and wing length of 34 males that could have been produced by queens (alate or dealate) or workers from 8 colonies and 86 worker-produced males from 13 queenless colonies. See Colony observations section for details on how male source was determined. Not all the measurements could be obtained from some males that were collected dead because of missing or damaged parts (e.g. missing head or indented alitrunk). Wing width and length were always measured using the same veins as reference (see Additional file 2: Fig. S2) to ensure consistency between individuals. Deformed wings were not measured as they did not show wing venation. All the colonies from which the samples originated were fed the same diet and were kept in the same rearing conditions (see Dissections of worker’s ovaries of entire colonies section for more details on the diet and rearing conditions).

#### Microsatellite analysis

We conducted microsatellite analysis at six markers to determine whether the observed heterozygosity of queen- and worker-produced males differed. We genotyped 20 males that could have been produced by queens (dealate or alate) or workers from 4 colonies and 55 worker-produced males from 12 colonies. We also genotyped 1–5 workers from 6 of these colonies with queen- or worker-produced males (*N* = 26 workers), 1–5 workers from 13 of the colonies with worker-produced males (*N* = 48), 1–3 queens from 4 of the colonies with queen- or worker-produced males to determine their observed heterozygosity (*n* = 9 queens in total, details of the genotyping protocol and a table summarising the characteristics of the microsatellite loci are in Additional file 2: Appendix S1). Individuals that were not successfully genotyped at all 6 loci were genotyped a second time and the run with the highest number of successful amplifications for each individual was kept. Individuals which were not successfully genotyped at 3 loci or more were not included in the analysis.

### Queen transfer experiment

#### Experimental design

We conducted a laboratory experiment to determine whether the absence of a queen triggers workers to become physogastric and lay eggs. We evenly split workers and brood from 14 colonies into two subcolonies and randomly assigned one of each pair to house a queen while the other was queenless. Each of the resulting 28 subcolonies had 121 to 200 workers depending on the size of the original colony (Additional file 1: Table S3). Each subcolony had several pieces of brood at the various stages present in the colony at that time. The colonies were collected from September to November 2017 at five sites in Queensland (Additional file 1: Table S3 and S4). Each subcolony was housed in a 150x220x320mm (height x width x depth) box with two 50 ml nesting tubes (length x diameter: 93x60mm) containing moist cotton and a 35x100x150mm piece of cardboard egg carton and maintained at 23.7 ± 0.78 °C (mean ± SD) and at ambient photoperiod. They were fed one mealworm biweekly and were provided with 25% sugar water ad libitum.

Eight queens (out of 14) died before or on day 60 while six queens survived past day 60 and were moved to their corresponding queenless subcolony pair to determine whether the queen would be accepted, and whether moving the original queen back to the queenless treatment reduced the percentage of physogastric workers and stopped workers from laying eggs. When moving the queen between subcolonies, we recorded worker behaviour towards the queen every 10 min for 1 h and recorded the queen’s position within the colony box after 24 h. We scored behaviour toward the queen according to Lai et al. (2015) [35] as: 0 ignoring, 1 touching, 2 avoiding, 3 holding, 4 aggression, and 5 fighting. At day 120 for all but the six queenless subcolonies with the most workers, we dissected the ovaries of 15 workers selected haphazardly among all the remaining workers, or all the workers if the subcolony was smaller than 15 workers. We continued monitoring six queenless colonies with the most remaining workers for another 60 days to determine whether they would produce males.

#### Colony observations

We counted the number of dead workers, brood (eggs, larvae, pupae) and determined whether trophic eggs were present (sub-spherically shaped eggs, [21]) weekly, and recorded the position (outside, under the egg carton or inside the nesting tubes) of physogastric and normal workers every two weeks to determine whether the behaviour of physogastric and normal workers differ.

### Aggression tests

We conducted aggression trials between *A. gracilipes* and native green tree ant (*Oecophylla smaragdina*) workers to determine whether *A. gracilipes* worker aggression toward a competitor differed depending on physogastric state (physogastric or normal) and colony state (queenless or queenright) in a fully factorial design. *Oecophylla smaragdina* is a native dominant species that had similar competitive ability of *A. gracilipes* in aggression tests with various *A. gracilipes*: *O. smaragdina* worker ratios in Borneo [56], although *A. gracilipes* displaces *O. smaragdina* in northern Australia [57]*.* Workers from queenless *A. gracilipes* colonies originated from the six colonies we had kept monitoring for 60 days after the end of the queen transfer experiment (Additional file 1: Table S3). These colonies were collected around Cairns and had been queenless for three to seven months. We did not have remaining queenright colonies from the queen transfer experiment, so we used six laboratory queenright colonies also collected around Cairns at the same dates as the queenless colonies (Additional file 1: Table S3) to avoid variation caused by time spent in the laboratory and colony origin. Workers from these colonies were approximately 4 mm in length. We collected *O. smaragdina* workers before each trial from a single tree on the James Cook University campus in Cairns to eliminate variation in *O. smaragdina* worker aggression due to workers originating from a different colony. We only selected minor workers (approximately 8 mm in length) that exhibited defensive behaviour (i.e. lifting their gaster to spray acid) when approached by our forceps. We let the *O. smaragdina* workers acclimatize to the laboratory for 10 min after collection. We used each *A. gracilipes* and *O. smaragdina* worker only once.

We measured aggressive interactions between three *A. gracilipes* workers that were either all physogastric or normal and a single *O. smaragdina* worker. We conducted a pilot experiment in which *O. smaragdina* workers always overcame *A. gracilipes* in 1:1 or 1:2 interactions. We therefore decided on a 1:3 ratio to enable us to detect differences between the aggression level and survival of physogastric and normal *A. gracilipes* workers. We replicated the aggression tests three times for both physogastric states (normal or physogastric) for each of the six queenless colonies and six queenright colonies. Only three normal workers were present in one queenless colony. Therefore, we ran only one aggression test between normal workers and one *O. smaragdina* worker for this colony. Thus, we ran 18 tests each of normal workers and physogastric workers from queenright colonies and physogastric workers from queenless colonies, and 16 tests for normal workers from queenless colonies (*n* = 70 tests in total).

We conducted the 60-min aggression tests in fluon-coated 60x93mm (diameter x height) PVC cylinders separated into two halves with a laminated paper card. The arenas were placed on a plastic tray that was washed with non-scented soap after each trial. We placed one *O. smaragdina* worker on one side and three *A. gracilipes* workers on the other side and let the ants acclimatize for five minutes before removing the dividing wall. For the first five minutes, we noted the highest aggression score between the two species at 30s intervals according to the method used in Lai et al. (2015) [35] and described above. We then checked the arena every 5 min for the remaining 55 min and recorded whether *O. smaragdina* or *A. gracilipes* workers had died. At the end of the trial, we collected the three *A. gracilipes* workers (dead or alive), placed them in ethanol, and dissected their ovaries.

### Data analysis

We analysed our data in R version 3.5.0 [58] and used functions from the stats package (R Development Core Team 2009) unless specified otherwise. All the models used are summarised in Table 3. We used generalized linear model (GLM, glm function) followed by likelihood ratio tests (Anova function in the car package [59] and generalized linear mixed model (GLMM, glmer function in the lme4 package [60], followed by Type III Wald χ2 tests (Anova function). We also used linear mixed-effects model (LMM, lmer function in the lme4 package) followed by Type II Wald χ2 tests and cumulative link mixed models (CLMM, clmm2 function in the package ordinal, [61] followed by likelihood ratio tests [62]. We used post-hoc Tukey tests to make pairwise comparisons (emmeans function, in the package emmeans, [63]) and tested the data for overdispersion where appropriate. We used an observation-level random intercept to re-evaluate models with over-dispersion in the queen transfer experiment and aggression tests and also changed the distribution to negative binomial for one of the aggression tests models (Table 3) because the first method was not sufficient to resolve overdispersion issues. We confirmed that the final GLM, GLMM, and LMM did not have heteroscedasticity and zero-inflation issues using the DHARMa package [64]. For the queen transfer experiment, we analysed data from replicates in which the queens survived until or past day 60 (10 queenright and 10 queenless colonies) separately from data obtained from replicates in which the queens survived until day 120 (3 queenright and 3 queenless colonies) because of the low queen survival rate at day 120.

**Table 3:** Summary table of final models and parameters. ‘x’ represents the interaction terms. Explanatory variables are fixed unless specified otherwise. Colony status: queenless or queenright, worker type: physogastric or normal *A. gracilipes*

Section	Model and distribution	Response variable	Explanatory variables
Dissections of worker’s ovaries of entire colonies	GLM binomial	Presence or absence of mature oocytes	Colony status Worker type
	GLM binomial	Worker type	Colony status
Colony observations	GLMM binomial	Whether the colony ever produced males	Whether the colony ever had a queen Maximum number of workers Whether the colony ever had a queen x Max. number of workers Number of days in captivity (random)
Morphometry and genotyping	LMM	Head width or Weber’s length or wing width or wing length	Male source Colony of origin (random)
Queen transfer experiment	GLMM binomial	Proportion of normal workers until day 60	Time^a^ Colony status Time^a^ x Colony status Colony of origin (random)
	GLMM binomial	Proportion of normal workers until day 120	Time^a^ Colony status Time^a^ x Colony status Colony of origin (random)
	GLMM binomial	Presence or absence of workers in the foraging area	Worker type Colony status Colony of origin (random)
	GLMM binomial	Presence absence mature oocytes	Colony status Worker type Colony of origin (random)
Aggression tests	CLMM	Maximal aggression score	Colony status Worker type Colony status x Worker type Colony of origin (random)
	GLMM negative binomial	Survival of all three *A. gracilipes* workers	Colony status Worker type Colony status x Worker type Colony of origin (random)
	GLMM binomial	Survival of the *O. smaragdina* worker	Colony status Worker type Colony status x Worker type Colony of origin (random)
	GLM binomial	Species initiating the fight	Colony status Worker type Colony status x Worker type
	GLMM binomial	Presence absence of mature oocytes	Colony status Worker type Colony of origin (random)

^a^ days since the start of the queen transfer experiment

## Supplementary Information

**Additional file 1 **: **Table S1**. Genotyping results of males, workers, and queens. **Table S2**. Site coordinates of where colonies were collected. **Table S3**. Additional information on the colonies used in dissections of worker’s ovaries of entire colonies, the queen transfer experiment, and the aggression tests. **Table S4** Additional information on the colonies used in the colony observations.

**Additional file 2 **: **Figure S1**. Boxplot showing the proportion of normal workers in the queen transfer experiment by colony status and number of days since the start of the experiment until day 120. **Figure S2**. Forewing of a male *A. gracilipes* showing which veins were used to measure wing width and wing length **Appendix S1**. Additional information on the genotyping protocol including **Table S5** which summarises the characteristic of the microsatellite loci.

## Data Availability

The datasets supporting the conclusions of this article are available in the Research Data (Tropical Data Hub) repository at James Cook University at  10.25903/sjzv-my64.
