# Supplementary material for: Origin, behaviour, and genetics of reproductive workers in an invasive ant
Source: Front Zool. 2021 Mar 22;18:13. doi: 10.1186/s12983-021-00392-2 (PMC7986258; doi:10.1186/s12983-021-00392-2)
Supplement: Supplementary file 1 — Additional file 1 : Table S1. Genotyping results of males, workers, and queens. Table S2. Site coordinates of where colonies were collected. Table S3. Additional information on the colonies used in dissections of worker’s ovaries of entire colonies, the queen transfer experiment, and the aggression tests. Table S4 Additional information on the colonies used in the colony observations. [file 12983_2021_392_MOESM1_ESM.docx]

**Additional file 1**

**Table S1.** Genotyping results. Male source: 1: dealate queen- or worker-produced, 2: worker-produced, 3: alate queen- or worker-produced. *-*: amplification failure

| **Colony code** | **Male source** | **Caste** | **Ano1** | | **Ano3** | | **Ano4** | | **Ano5** | | **Ano6** | | **Ano9** | | **Notes on deformity** |
| --- | --- | --- | --- | --- | --- | --- | --- | --- | --- | --- | --- | --- | --- | --- | --- |
| 0711WC3 | 1 | Male | 99 | 99 | 140 | 140 | - | - | - | - | - | - | 155 | 155 |  |
| 0711WC3 | 1 | Male | 103 | 103 | - | - | 156 | 156 | 122 | 122 | - | - | 155 | 155 |  |
| 0711WC3 | 1 | Male | 103 | 103 | - | - | 156 | 156 | 122 | 122 | - | - | - | - |  |
| 0711WC3 | 1 | Male | - | - | 160 | 160 | 156 | 156 | 122 | 122 | - | - | - | - |  |
| 0711WC3 | 1 | Male | - | - | - | - | 156 | 156 | - | - | 114 | 114 | 149 | 149 |  |
| 0711WC3 | 1 | Male | 103 | 103 | 160 | 160 | - | - | - | - | - | - | 149 | 149 |  |
| 0711WC3 | 1 | Male | - | - | 160 | 160 | 156 | 156 | - | - | - | - | 149 | 149 |  |
| 0711WC3 | 1 | Male | 103 | 103 | 160 | 160 | 156 | 164 | 122 | 122 | - | - | 149 | 149 |  |
| 0711WC3 | 1 | Worker | 99 | 103 | - | - | 156 | 174 | - | - | - | - | 149 | 155 |  |
| 0711WC3 | 1 | Queen | 103 | 103 | 160 | 160 | 156 | 156 | 122 | 122 | - | - | 149 | 149 |  |
| 0711WC3 | 1 | Queen | 103 | 103 | 160 | 160 | 156 | 156 | 122 | 122 | 116 | 116 | 149 | 149 |  |
| 0711WC3 | 1 | Queen | 103 | 103 | 160 | 160 | 156 | 156 | 122 | 122 | 116 | 116 | 153 | 153 |  |
| 1011RPT3S10 | 1 | Queen | - | - | 160 | 160 | 156 | 174 | 118 | 118 | - | - | 153 | 153 |  |
| 1011RPT4S7 | 1 | Worker | 101 | 105 | 160 | 166 | 156 | 174 | 118 | 120 | 116 | 130 | 153 | 155 |  |
| 1011RPT4S7 | 1 | Worker | 101 | 105 | 160 | 166 | 156 | 174 | 118 | 120 | 116 | 130 | 153 | 155 |  |
| 1011RPT4S7 | 1 | Worker | 101 | 105 | 160 | 166 | 156 | 174 | 118 | 120 | 116 | 130 | 153 | 155 |  |
| 1011RPT4S7 | 1 | Worker | 101 | 105 | 160 | 166 | - | - | 118 | 120 | 116 | 130 | 153 | 155 |  |
| 1011RPT4S7 | 1 | Worker | 101 | 105 | 160 | 166 | 156 | 174 | 118 | 120 | 116 | 130 | 153 | 155 |  |
| 1011RPT4S7 | 1 | Queen | 105 | 105 | - | - | - | - | 120 | 120 | 116 | 116 | 153 | 153 |  |
| 1011RPT4S7 | 1 | Queen | 105 | 105 | 160 | 160 | 156 | 156 | 118 | 118 | 116 | 116 | 153 | 153 |  |
| 1011RPT4S7 | 1 | Queen | 105 | 105 | 160 | 160 | - | - | 120 | 120 | 116 | 116 | 153 | 153 |  |
| 20170316GCT4-9 | 1 | Male | 105 | 105 | 160 | 160 | - | - | 118 | 118 | - | - | 153 | 153 |  |
| 20170316GCT4-9 | 1 | Worker | 101 | 105 | 160 | 166 | 156 | 174 | 118 | 120 | 116 | 130 | 153 | 155 |  |
| 20170316GCT4-9 | 1 | Worker | 101 | 105 | 160 | 166 | 156 | 174 | 118 | 120 | 116 | 130 | 153 | 155 |  |
| 20170316GCT4-9 | 1 | Worker | 101 | 105 | 160 | 166 | 156 | 174 | 118 | 120 | 116 | 130 | 153 | 155 |  |
| 20170316GCT4-9 | 1 | Worker | 101 | 105 | 160 | 166 | 156 | 174 | 118 | 120 | 116 | 130 | 153 | 155 |  |
| 20170316GCT4-9 | 1 | Worker | 101 | 105 | 160 | 166 | 156 | 174 | 118 | 120 | 116 | 130 | 153 | 155 |  |
| 20170717DCT2-1 | 1 | Male | 105 | 105 | 160 | 160 | 156 | 156 | 120 | 120 | - | - | 155 | 155 |  |
| 20170717DCT2-1 | 1 | Male | 105 | 105 | 160 | 160 | 156 | 156 | 120 | 120 | - | - | 155 | 155 |  |
| 20170717DCT2-1 | 1 | Male | 105 | 105 | 160 | 160 | 156 | 156 | 120 | 120 | 116 | 116 | 155 | 155 |  |
| 20170717DCT2-1 | 1 | Male | 101 | 105 | 160 | 168 | 156 | 174 | 120 | 120 | - | - | 155 | 155 |  |
| 20170717DCT2-1 | 1 | Male | 105 | 105 | 160 | 160 | 156 | 156 | 120 | 120 | - | - | 155 | 155 |  |
| 20170717DCT2-1 | 1 | Worker | - | - | 160 | 168 | 156 | 174 | 118 | 120 | 116 | 130 | - | - |  |
| 20170717DCT2-1 | 1 | Worker | 101 | 105 | 160 | 168 | 156 | 174 | 118 | 120 | 116 | 130 | 153 | 155 |  |
| 20170717DCT2-1 | 1 | Worker | 101 | 105 | 160 | 168 | - | - | 118 | 120 | 116 | 130 | 153 | 155 |  |
| 20170717DCT2-1 | 1 | Worker | 101 | 105 | 160 | 168 | 156 | 174 | 118 | 120 | 116 | 130 | 153 | 155 |  |
| 20170717DCT2-1 | 1 | Worker | 101 | 105 | 160 | 168 | 156 | 174 | 118 | 120 | 116 | 130 | 153 | 155 |  |
| 20170717DCT2-1 | 1 | Queen | 105 | 105 | 160 | 160 | 156 | 156 | 120 | 120 | 116 | 116 | 155 | 155 |  |
| 0308RPT4S6 | 2 | Male | 105 | 105 | 166 | 166 | 174 | 174 | 116 | 116 | 114 | 114 | 155 | 155 |  |
| 0407Kearns13 | 2 | Male | 105 | 105 | 166 | 166 | - | - | 122 | 122 | - | - | 153 | 153 | deformed wings |
| 0407Kearns13 | 2 | Male | 105 | 105 | 160 | 160 | - | - | 122 | 122 | - | - | 155 | 155 |  |
| 0407Kearns13 | 2 | Male | 99 | 99 | 160 | 160 | - | - | 118 | 118 | - | - | - | - |  |
| 0407Kearns13 | 2 | Male | - | - | - | - | 156 | 156 | 118 | 118 | - | - | 155 | 155 |  |
| 0407Kearns13 | 2 | Male | 105 | 105 | - | - | 156 | 156 | - | - | 114 | 114 | - | - |  |
| 0407Kearns13 | 2 | Male | 105 | 105 | - | - | - | - | 118 | 118 | 128 | 128 | - | - |  |
| 0407Kearns13 | 2 | Male | 105 | 105 | 166 | 166 | - | - | 118 | 118 | - | - | - | - |  |
| 0407Kearns13 | 2 | Male | 105 | 105 | 166 | 166 | - | - | 118 | 118 | 114 | 114 | - | - |  |
| 0407Kearns13 | 2 | Male | - | - | - | - | 174 | 174 | - | - | 114 | 114 | 155 | 155 |  |
| 0407Kearns13 | 2 | Male | 99 | 99 | - | - | - | - | 116 | 116 | 114 | 114 | - | - |  |
| 0407Kearns13 | 2 | Male | 105 | 105 | - | - | - | - | - | - | 114 | 114 | 153 | 153 |  |
| 0407Kearns13 | 2 | Male | 105 | 105 | 166 | 166 | 156 | 156 | - | - | 114 | 114 | - | - |  |
| 0407Kearns13 | 2 | Male | - | - | 166 | 166 | 156 | 156 | - | - | - | - | 155 | 155 |  |
| 0407Kearns13 | 2 | Worker | 99 | 107 | - | - | 156 | 174 | - | - | 114 | 128 | 153 | 155 |  |
| 0407Kearns13 | 2 | Worker | 99 | 105 | 166 | 166 | 156 | 174 | - | - | 114 | 128 | 153 | 155 |  |
| 0407Kearns13 | 2 | Worker | 99 | 105 | 166 | 166 | 156 | 174 | - | - | 114 | 128 | 153 | 155 |  |
| 0407Kearns13 | 2 | Worker | 99 | 105 | - | - | 156 | 174 | - | - | 114 | 128 | 153 | 155 |  |
| 0407Kearns13 | 2 | Worker | 99 | 105 | 160 | 166 | 156 | 174 | - | - | 114 | 128 | 153 | 155 |  |
| 0407Kearns15 | 2 | Male | 105 | 105 | 166 | 166 | 174 | 174 | - | - | - | - | 155 | 155 |  |
| 0407Kearns15 | 2 | Worker | 99 | 105 | 166 | 166 | 156 | 174 | - | - | 114 | 128 | 153 | 153 |  |
| 0407Kearns15 | 2 | Worker | - | - | 166 | 166 | 156 | 174 | - | - | 114 | 128 | - | - |  |
| 0407Kearns15 | 2 | Worker | 99 | 105 | 166 | 166 | 156 | 174 | - | - | 114 | 128 | - | - |  |
| 0407Kearns15 | 2 | Worker | 99 | 105 | 166 | 166 | 156 | 174 | - | - | 114 | 128 | 153 | 155 |  |
| 0407Kearns15 | 2 | Worker | 99 | 105 | 166 | 166 | 156 | 174 | - | - | 114 | 128 | 153 | 155 |  |
| 0407RPT3S4 | 2 | Male | 99 | 99 | 160 | 160 | 156 | 174 | 118 | 118 | - | - | 153 | 153 |  |
| 0407RPT3S4 | 2 | Male | - | - | 166 | 166 | 156 | 156 | 118 | 118 | 128 | 128 | 153 | 153 |  |
| 0407RPT3S4 | 2 | Male | - | - | 166 | 166 | 156 | 174 | 118 | 118 | 128 | 128 | 155 | 155 |  |
| 0407RPT3S4 | 2 | Male | - | - | 166 | 166 | 174 | 174 | 116 | 116 | - | - | - | - |  |
| 0407RPT3S4 | 2 | Male | - | - | - | - | - | - | 116 | 116 | 114 | 114 | 153 | 153 |  |
| 0407RPT3S4 | 2 | Worker | 99 | 105 | - | - | 156 | 174 | - | - | 114 | 128 | 153 | 153 |  |
| 0407RPT3S4 | 2 | Worker | 99 | 105 | 166 | 166 | 156 | 174 | - | - | 114 | 128 | 153 | 153 |  |
| 0407RPT3S4 | 2 | Worker | 99 | 105 | 160 | 166 | 156 | 174 | - | - | - | - | 153 | 153 |  |
| 0407RPT3S4 | 2 | Worker | 99 | 105 | 160 | 166 | 156 | 174 | - | - | - | - | 153 | 153 |  |
| 0407RPT3S4 | 2 | Worker | 99 | 105 | 160 | 166 | 156 | 174 | - | - | 114 | 128 | 153 | 155 |  |
| 0606Kearns17 | 2 | Worker | 99 | 105 | 166 | 166 | 156 | 174 | - | - | 114 | 128 | 153 | 155 |  |
| 0606Kearns17 | 2 | Worker | - | - | 166 | 166 | - | - | - | - | 114 | 128 | 153 | 153 |  |
| 0606RPT3S3 | 2 | Male | 99 | 99 | 166 | 166 | 174 | 174 | 118 | 118 | - | - | - | - |  |
| 0606RPT3S3 | 2 | Worker | 99 | 105 | 166 | 166 | 156 | 174 | - | - | 114 | 128 | 153 | 155 |  |
| 0606RPT3S3 | 2 | Worker | 99 | 105 | 160 | 166 | 156 | 174 | - | - | 114 | 128 | 153 | 155 |  |
| 0606RPT3S3 | 2 | Worker | 99 | 105 | 160 | 166 | 156 | 174 | - | - | 114 | 128 | 153 | 155 |  |
| 0606RPT3S3 | 2 | Worker | - | - | 160 | 166 | 156 | 174 | - | - | 114 | 128 | 155 | 155 |  |
| 0606RPT3S5 | 2 | Male | 99 | 99 | 166 | 166 | 156 | 174 | - | - | 114 | 114 | 153 | 153 |  |
| 0606RPT3S5 | 2 | Male | 105 | 105 | 160 | 160 | - | - | - | - | 114 | 114 | 153 | 153 |  |
| 0606RPT3S5 | 2 | Male | 105 | 105 | - | - | - | - | 118 | 118 | 114 | 114 | - | - |  |
| 0606RPT3S5 | 2 | Male | 105 | 105 | 160 | 160 | 174 | 174 | 118 | 118 | - | - | - | - |  |
| 0606RPT3S5 | 2 | Worker | 99 | 99 | 160 | 166 | 156 | 174 | - | - | 114 | 128 | 155 | 155 |  |
| 0606RPT3S5 | 2 | Worker | 99 | 105 | 160 | 166 | 156 | 174 | - | - | 114 | 128 | 153 | 153 |  |
| 0606RPT3S5 | 2 | Worker | 99 | 105 | 160 | 166 | 156 | 174 | - | - | 114 | 128 | 153 | 155 |  |
| 0606RPT3S5 | 2 | Worker | 99 | 105 | 160 | 166 | 156 | 174 | - | - | 114 | 128 | 155 | 155 |  |
| 0606SC10 | 2 | Male | 105 | 105 | 166 | 166 | 174 | 174 | 116 | 116 | - | - | 155 | 155 |  |
| 0606SC10 | 2 | Male | 99 | 99 | - | - | 174 | 174 | 118 | 118 | 128 | 128 | - | - |  |
| 0606SC10 | 2 | Male | 105 | 105 | - | - | 174 | 174 | 116 | 116 | - | - | - | - |  |
| 0606SC10 | 2 | Worker | 99 | 105 | - | - | 156 | 174 | - | - | 114 | 128 | 153 | 155 |  |
| 0606SC10 | 2 | Worker | - | - | 160 | 166 | 156 | 174 | - | - | 114 | 128 | 153 | 155 |  |
| 0606SC10 | 2 | Worker | 99 | 105 | - | - | 156 | 174 | - | - | - | - | 153 | 155 |  |
| 0606SC10 | 2 | Worker | 99 | 105 | - | - | 156 | 174 | - | - | 114 | 128 | 153 | 155 |  |
| 0712RPT2S6 | 2 | Male | 99 | 99 | 166 | 166 | - | - | 118 | 118 | - | - | 155 | 155 |  |
| 0712RPT2S6 | 2 | Male | 99 | 99 | 166 | 166 | 174 | 174 | 118 | 118 | - | - | 153 | 153 |  |
| 0712RPT2S6 | 2 | Male | 99 | 99 | 160 | 160 | - | - | 116 | 116 | - | - | 153 | 153 |  |
| 0712RPT2S6 | 2 | Male | 105 | 105 | 160 | 160 | 174 | 174 | 118 | 118 | - | - | 153 | 153 |  |
| 0712RPT2S6 | 2 | Male | 105 | 105 | 160 | 160 | 156 | 156 | - | - | - | - | - | - | deformed wings |
| 0712RPT2S6 | 2 | Male | 99 | 99 | 166 | 166 | 156 | 156 | 116 | 116 | - | - | - | - |  |
| 0712RPT2S6 | 2 | Male | 99 | 99 | 160 | 160 | 156 | 156 | - | - | - | - | - | - |  |
| 0712RPT2S6 | 2 | Male | 99 | 99 | 166 | 166 | - | - | 116 | 116 | - | - | 155 | 155 |  |
| 0712RPT2S6 | 2 | Worker | 99 | 105 | 160 | 166 | 156 | 174 | - | - | 114 | 128 | 155 | 155 |  |
| 0712RPT2S6 | 2 | Worker | 99 | 105 | 160 | 166 | 156 | 174 | - | - | 114 | 128 | 153 | 155 |  |
| 0712RPT2S6 | 2 | Worker | 99 | 105 | 166 | 166 | 156 | 174 | - | - | 114 | 128 | 153 | 155 |  |
| 0712RPT2S6 | 2 | Worker | 99 | 105 | 160 | 166 | 156 | 174 | - | - | 114 | 128 | 153 | 155 |  |
| 0712RPT2S6 | 2 | Worker | 99 | 105 | 160 | 166 | 156 | 174 | - | - | 114 | 128 | 153 | 155 |  |
| 1108Kearns6 | 2 | Male | 105 | 105 | - | - | 174 | 174 | - | - | 128 | 128 | - | - |  |
| 1108Kearns6 | 2 | Male | 99 | 99 | 166 | 166 | 174 | 174 | 118 | 118 | 114 | 114 | - | - |  |
| 1108Kearns6 | 2 | Male | 105 | 105 | 166 | 166 | 174 | 174 | 118 | 118 | 114 | 114 | 153 | 153 |  |
| 1108Kearns6 | 2 | Worker | - | - | - | - | 156 | 174 | - | - | 114 | 128 | 153 | 155 |  |
| 1108Kearns6 | 2 | Worker | 99 | 105 | 166 | 166 | 156 | 174 | - | - | 114 | 128 | 153 | 155 |  |
| 1108Kearns6 | 2 | Worker | 99 | 105 | - | - | 156 | 174 | - | - | 114 | 128 | 153 | 155 |  |
| 1108Kearns6 | 2 | Worker | 99 | 105 | - | - | 156 | 174 | - | - | 114 | 128 | 153 | 155 |  |
| 1108Kearns6 | 2 | Worker | 99 | 105 | 166 | 166 | 156 | 174 | - | - | 114 | 128 | 155 | 155 |  |
| 1201Kearns20 | 2 | Male | 99 | 99 | 166 | 166 | - | - | 116 | 116 | - | - | 153 | 153 |  |
| 1201Kearns20 | 2 | Male | 99 | 99 | 166 | 166 | 156 | 156 | 116 | 116 | - | - | - | - |  |
| 1201Kearns20 | 2 | Worker | 99 | 107 | 166 | 166 | 156 | 174 | - | - | - | - | 153 | 155 |  |
| 1201Kearns20 | 2 | Worker | 99 | 105 | 166 | 166 | 156 | 174 | - | - | 114 | 128 | 153 | 155 |  |
| 1201RPT3S6 | 2 | Male | 99 | 99 | 166 | 166 | - | - | 118 | 118 | - | - | 155 | 155 | one eye bigger |
| 1201RPT3S6 | 2 | Male | 105 | 105 | 160 | 160 | - | - | 118 | 118 | - | - | - | - | deformed wings |
| 1201RPT3S6 | 2 | Male | 99 | 99 | 160 | 160 | - | - | 118 | 118 | - | - | 155 | 155 |  |
| 1201RPT3S6 | 2 | Male | 105 | 105 | 166 | 166 | - | - | 118 | 118 | - | - | - | - |  |
| 1201RPT3S6 | 2 | Male | 105 | 105 | 166 | 166 | 156 | 156 | - | - | - | - | - | - |  |
| 1201RPT3S6 | 2 | Worker | - | - | 166 | 166 | 156 | 174 | - | - | 114 | 128 | 153 | 155 |  |
| 1201RPT3S6 | 2 | Worker | 99 | 105 | 160 | 166 | 156 | 174 | - | - | 114 | 128 | 153 | 155 |  |
| 1201RPT3S6 | 2 | Worker | 99 | 105 | 160 | 166 | 156 | 174 | - | - | 114 | 128 | 153 | 155 |  |
| 1201RPT3S6 | 2 | Worker | 99 | 105 | 160 | 166 | 156 | 174 | - | - | 114 | 128 | 153 | 155 |  |
| 1502Kearns13 | 2 | Male | 99 | 99 | - | - | 174 | 174 | - | - | 128 | 128 | 153 | 153 |  |
| 1502Kearns13 | 2 | Male | 99 | 99 | - | - | 156 | 156 | 118 | 118 | 114 | 114 | - | - |  |
| 1502Kearns13 | 2 | Male | 99 | 99 | - | - | 174 | 174 | 116 | 116 | 128 | 128 | - | - |  |
| 1502Kearns13 | 2 | Male | 99 | 99 | 166 | 166 | 174 | 174 | 118 | 118 | 114 | 114 | - | - |  |
| 1502Kearns13 | 2 | Male | - | - | - | - | 156 | 156 | - | - | 114 | 114 | 153 | 153 |  |
| 1502Kearns13 | 2 | Male | - | - | 166 | 166 | - | - | - | - | 128 | 128 | 155 | 155 |  |
| 1502Kearns13 | 2 | Male | - | - | 166 | 166 | 156 | 156 | - | - | - | - | 153 | 153 |  |
| 1502Kearns13 | 2 | Male | - | - | 166 | 166 | 156 | 156 | - | - | - | - | 153 | 153 |  |
| 1502Kearns13 | 2 | Male | 105 | 105 | - | - | 156 | 156 | - | - | - | - | 155 | 155 |  |
| 1502Kearns13 | 2 | Worker | 99 | 105 | - | - | 156 | 174 | - | - | - | - | 153 | 153 |  |
| 1502Kearns13 | 2 | Worker | 99 | 105 | - | - | 156 | 174 | - | - | 114 | 128 | 153 | 153 |  |
| 2110Kearns3 | 2 | Worker | 99 | 99 | - | - | 156 | 174 | - | - | 114 | 128 | - | - |  |
| 0606RPT2S3 | 3 | Male | 105 | 105 | 166 | 166 | 174 | 174 | 118 | 118 | 114 | 114 | - | - |  |
| 0606RPT2S3 | 3 | Male | 99 | 99 | - | - | 174 | 174 | 118 | 118 | - | - | - | - |  |
| 0606RPT2S3 | 3 | Male | 99 | 99 | 166 | 166 | 156 | 156 | - | - | - | - | 155 | 155 |  |
| 0606RPT2S3 | 3 | Male | 99 | 99 | 160 | 160 | - | - | - | - | 114 | 114 | 153 | 153 |  |
| 0606RPT2S3 | 3 | Male | - | - | 160 | 160 | 156 | 156 | - | - | - | - | 153 | 153 |  |
| 0606RPT2S3 | 3 | Worker | 99 | 105 | 160 | 166 | 156 | 174 | - | - | 114 | 128 | 153 | 153 |  |
| 0606RPT2S3 | 3 | Worker | 99 | 105 | - | - | 156 | 174 | - | - | - | - | 153 | 153 |  |
| 0606RPT2S3 | 3 | Worker | 99 | 105 | 160 | 166 | 156 | 174 | - | - | - | - | 153 | 153 |  |
| 0606RPT2S3 | 3 | Worker | 99 | 105 | 160 | 166 | 156 | 174 | - | - | - | - | 153 | 153 |  |
| 0606RPT2S3 | 3 | Worker | 99 | 105 | 160 | 166 | 156 | 174 | - | - | 114 | 128 | 153 | 153 |  |
| 0606RPT2S3 | 3 | Queen | 105 | 105 | 160 | 160 | 156 | 156 | 120 | 120 | 116 | 116 | 153 | 153 |  |
| 1201RPT3S10 | 3 | Male | - | - | 166 | 166 | 174 | 174 | 118 | 118 | - | - | - | - |  |
| 1201RPT3S10 | 3 | Worker | - | - | 160 | 166 | 156 | 174 | - | - | 114 | 128 | 153 | 153 |  |
| 1201RPT3S10 | 3 | Worker | 99 | 105 | - | - | 156 | 174 | - | - | 114 | 128 | 153 | 153 |  |
| 1201RPT3S10 | 3 | Worker | 99 | 105 | 160 | 166 | 156 | 174 | - | - | 114 | 128 | 153 | 153 |  |
| 1201RPT3S10 | 3 | Worker | 99 | 105 | - | - | 156 | 174 | - | - | 114 | 128 | 153 | 153 |  |
| 1201RPT3S10 | 3 | Worker | 99 | 105 | 160 | 166 | 156 | 174 | - | - | 114 | 128 | 153 | 153 |  |

**Table S2.** Site coordinates

| **Site** | **Latitude** | **Longitude** |
| --- | --- | --- |
| Kearns | -17.004 | 145.717 |
| Russet Park | -16.803 | 145.597 |
| Sandy Creek | -17.056 | 145.734 |
| Mount Peter | -17.050 | 145.728 |
| Grey's Creek | -17.062 | 145.741 |
| Vindula | -17.052 | 145.729 |
| Fantin Creek | -17.076 | 145.743 |
| Gordonvale | -17.083 | 145.796 |
| Gordonvale Mill | -17.093 | 145.789 |
| Hussey | -17.064 | 145.740 |
| White Creek | -19.368 | 146.955 |
| Draper Creek | -17.083 | 145.756 |
| Frank’s Creek | -17.045 | 145.728 |
| Frank’s Rainforest | -17.046 | 145.725 |
| Harold | -17.055 | 145.729 |
| Swallow | -17.003 | 145.750 |
| Whereat | -17.022 | 145.712 |
| Sikh Temple | -17.038 | 145.751 |
| Mt Sheridan | -16.995 | 145.727 |
| Wrights Creek1 | -17.066 | 145.745 |
| Falcon | -16.967 | 145.726 |
| Green Forest Road | -16.817 | 145.577 |
| Townsville | -19.259 | 146.817 |
| Hervey Bay | -25.288 | 152.768 |
| Brisbane | -27.391 | 153.118 |
| Nome | -19.374 | 146.907 |

**Table S3.** Details of colonies used for 1: Dissections of worker’s ovaries of entire colonies, 2: Queen transfer experiment, and 3: Aggression tests. Last column shows the number of workers per replicate at the start of the queen transfer experiment. NA: Not applicable. Some colonies were used for more than one purpose.

| **Collection date** | **Collection Site** | **Colony code** | **Used for** | **Status** | **Workers per replicate at day 0** |
| --- | --- | --- | --- | --- | --- |
| 20171020B4 | Brisbane | Oct-17 | 1 | Queenright | NA |
| 20171020B4 | Brisbane | Oct-17 | 2 | Queenright | 170 |
| 20171101D3 | Draper Creek | Nov-17 | 3 | Queenright | NA |
| 20171101DC2 | Draper Creek | Nov-17 | 3 | Queenright | NA |
| 20171109DC1 | Draper Creek | Nov-17 | 3 | Queenright | NA |
| 20171113DC1 | Draper Creek | Nov-17 | 3 | Queenright | NA |
| 20170910GV1 | Gordonvale | Sep-17 | 2 | Queenright | 200 |
| 20170910GV2 | Gordonvale | Sep-17 | 2 | Queenright | 200 |
| 20170910GV2 | Gordonvale | Sep-17 | 3 | Queenless | NA |
| 20171109GV1 | Gordonvale | Nov-17 | 2 | Queenright | 200 |
| 20171109GV1 | Gordonvale | Nov-17 | 3 | Queenless | NA |
| 20171109GV2 | Gordonvale | Nov-17 | 3 | Queenless | NA |
| 20171113GV1 | Gordonvale | Nov-17 | 2 | Queenright | 150 |
| 20171127GV1 | Gordonvale | Nov-17 | 1 | Queenless | NA |
| 20171127GV1 | Gordonvale | Nov-17 | 2 | Queenright | 200 |
| 20171127GV1 | Gordonvale | Nov-17 | 3 | Queenless | NA |
| GC200417 | Grey’s Creek | Apr-17 | 1 | Queenless | NA |
| 20171023HB2 | Hervey Bay | Oct-17 | 2 | Queenright | 200 |
| 20171014NomeTSV2 | Nome | Oct-17 | 2 | Queenright | 160 |
| 20171104NomeTSV2 | Nome | Nov-17 | 2 | Queenright | 135 |
| 20171104NomeTSV31 | Nome | Nov-17 | 2 | Queenright | 160 |
| 20171104NomeTSV4 | Nome | Nov-17 | 2 | Queenright | 200 |
| 20171104NomeTSV4 | Nome | Nov-17 | 2 | Queenright | 200 |
| 20171104Nome-TSV4 | Nome | Nov-17 | 1 | Queenright | NA |
| 20170910SC5 | Sandy Creek | Sep-17 | 2 | Queenright | 150 |
| 20170910SC5 | Sandy Creek | Sep-17 | 3 | Queenless | NA |
| 20170910SC5 | Sandy Creek | Sep-17 | 3 | Queenless | NA |
| 20170921SC3 | Sandy Creek | Sep-17 | 3 | Queenright | NA |
| 20170921SC9 | Sandy Creek | Sep-17 | 3 | Queenright | NA |
| 20171109SC1 | Sandy Creek | Nov-17 | 2 | Queenright | 121 |

**Table S4** Colony collection sites, status, time in captivity and number of workers and queens during the colony observations. Male source 1= worker produced; 2 =dealate queen or worker produced; 3= alate queen or worker produced.

| **Colony code** | **Collection site** | **Collection date** | **Days in captivity** | **Male producing** | **Male source** | **Number of workers** | **Number of queens** |
| --- | --- | --- | --- | --- | --- | --- | --- |
| 0308Franks20 | Frank’s Rainforest | Aug-16 | 244 | No |  | 0-25 | 0 |
| 0308Franks3 | Frank’s Rainforest | Aug-16 | 335 | No |  | 0-100 | 0-4 |
| 0308Franks5 | Frank’s Rainforest | Aug-16 | 273 | No |  | 0-100 | 0-8 |
| 0308Harold3 | Harold | Aug-16 | 278 | No |  | 0-46 | 0 |
| 0308Kearns12 | Kearns | Aug-16 | 314 | Yes | 1 | 0-327 | 0 |
| 0308Kearns13 | Kearns | Aug-16 | 209 | No |  | 0-165 | 0 |
| 0308Kearns2 | Kearns | Aug-16 | 293 | Yes | 1 | 0-387 | 0 |
| 0308Kearns7 | Kearns | Aug-16 | 321 | No |  | 0-196 | 0 |
| 0308RPT1-S6 | Russet Park | Aug-16 | 286 | No |  | 0-147 | 0 |
| 0308RPT2-S2 | Russet Park | Aug-16 | 118 | No |  | 0-90 | 0-1 |
| 0308RPT2-S6 | Russet Park | Aug-16 | 328 | No |  | 15-175 | 0 |
| 0308RPT3-S2 | Russet Park | Aug-16 | 278 | No |  | 5-85 | 0 |
| 0308RPT4-S4 | Russet Park | Aug-16 | 285 | No |  | 0-165 | 0 |
| 0308RPT4-S6 | Russet Park | Aug-16 | 328 | Yes | 1 | 5-275 | 0 |
| 0308Sandy13 | Sandy Creek | Aug-16 | 384 | No |  | 0-280 | 0-6 |
| 0308Sandy6 | Sandy Creek | Aug-16 | 286 | No |  | 0-285 | 0-6 |
| 0407Franks | Frank’s Rainforest | Jul-16 | 265 | No |  | 0-412 | 0-7 |
| 0407Kearns13 | Kearns | Jul-16 | 303 | Yes | 1 | 0-436 | 0 |
| 0407Kearns15 | Kearns | Jul-16 | 296 | Yes | 1 | 0-227 | 0 |
| 0407Kearns19 | Kearns | Jul-16 | 238 | Yes | 1,2 | 0-472 | 0 |
| 0407Kearns5 | Kearns | Jul-16 | 245 | No |  | 0-85 | 0 |
| 0407RPT1-S10 | Russet Park | Jul-16 | 260 | No |  | 0-275 | 0-2 |
| 0407RPT1-S2 | Russet Park | Jul-16 | 253 | No |  | 0-175 | 0 |
| 0407RPT2-S5 | Russet Park | Jul-16 | 260 | No |  | 0-206 | 0 |
| 0407RPT2-S8 | Russet Park | Jul-16 | 265 | No |  | 0-372 | 1-1 |
| 0407RPT3-S4 | Russet Park | Jul-16 | 303 | Yes | 1 | 15-227 | 0 |
| 0407RPT4-S1 | Russet Park | Jul-16 | 216 | No |  | 5-100 | 0 |
| 0407RPT4-S7 | Russet Park | Jul-16 | 260 | No |  | 0-175 | 0-1 |
| 0407SC16 | Sandy Creek | Jul-16 | 231 | No |  | 0-206 | 0-2 |
| 0407SC2 | Sandy Creek | Jul-16 | 322 | No |  | 0-206 | 0-4 |
| 0407SC3 | Sandy Creek | Jul-16 | 511 | No |  | 5-252 | 0-5 |
| 0407SC7 | Sandy Creek | Jul-16 | 296 | No |  | 0-175 | 0 |
| 0606Kearns1 | Kearns | Jun-16 | 252 | No |  | 0-306 | 0-1 |
| 0606Kearns17 | Kearns | Jun-16 | 273 | Yes | 1 | 0-337 | 0 |
| 0606Kearns3 | Kearns | Jun-16 | 324 | No |  | 0-430 | 0-2 |
| 0606RPT1-S2 | Russet Park | Jun-16 | 301 | Yes | 1 | 0-327 | 0 |
| 0606RPT1-S6 | Russet Park | Jun-16 | 266 | No |  | 0-296 | 0 |
| 0606RPT2-S10 | Russet Park | Jun-16 | 252 | No |  | 0-296 | 0-1 |
| 0606RPT2-S3 | Russet Park | Jun-16 | 266 | Yes | 3 | 0-291 | 0-1 |
| 0606RPT3-S3 | Russet Park | Jun-16 | 357 | Yes | 1 | 0-611 | 0 |
| 0606RPT3-S5 | Russet Park | Jun-16 | 259 | Yes | 1 | 0-260 | 0 |
| 0606RPT4-S9 | Russet Park | Jun-16 | 252 | No |  | 0-285 | 0 |
| 0606SC1 | Sandy Creek | Jun-16 | 244 | No |  | 0-221 | 0 |
| 0606SC10 | Sandy Creek | Jun-16 | 280 | Yes | 1 | 0-490 | 0 |
| 0606SC18 | Sandy Creek | Jun-16 | 204 | No |  | 0-196 | 0 |
| 0711WC1 | White Creek | Nov-16 | 274 | No |  | 0-110 | 0-5 |
| 0711WC2 | White Creek | Nov-16 | 490 | No |  | 0-170 | 0-12 |
| 0711WC3 | White Creek | Nov-16 | 134 | Yes | 2 | 0-350 | 0-7 |
| 0711WC4 | White Creek | Nov-16 | 90 | No |  | 0-165 | 0 |
| 0711WC5 | White Creek | Nov-16 | 205 | Yes | 2 | 0-350 | 0-3 |
| 0712GC13 | Grey’s Creek | Dec-16 | 201 | Yes | 2 | 5-280 | 0 |
| 0712GC2 | Grey’s Creek | Dec-16 | 201 | Yes | 2 | 5-280 | 0 |
| 0712RPT2-S6 | Russet Park | Dec-16 | 419 | Yes | 1,2 | 5-460 | 0 |
| 0712RPT4-S1 | Russet Park | Dec-16 | 202 | No |  | 5-165 | 0 |
| 0712RPT4-S8 | Russet Park | Dec-16 | 279 | Yes | 2,3 | 0-231 | 0-1 |
| 0809HerveyBay | Hervey Bay | Sep-16 | 193 | No |  | 0-46 | 0 |
| 0905MPRF1 | Mount Peter | May-16 | 259 | Yes | 1 | 0-175 | 0 |
| 0905MPRF2 | Mount Peter | May-16 | 287 | Yes | 3 | 0-316 | 0-2 |
| 0905MPRF4 | Mount Peter | May-16 | 203 | Yes | 2 | 0-366 | 0-2 |
| 1009Townsville | Townsville | Sep-16 | 261 | No |  | 0-72 | 0-5 |
| 1011RPT1-S2 | Russet Park | Nov-16 | 356 | No |  | 0-311 | 0-4 |
| 1011RPT4-S7 | Russet Park | Nov-16 | 306 | Yes | 2 | 0-350 | 0-4 |
| 1011RPT4-S9 | Russet Park | Nov-16 | 229 | Yes | 1 | 41-270 | 0 |
| 1108Kearns14 | Kearns | Aug-16 | 305 | No |  | 0-121 | 0 |
| 1108Kearns16 | Kearns | Aug-16 | 270 | No |  | 0-95 | 0 |
| 1108Kearns18 | Kearns | Aug-16 | 298 | No |  | 0-105 | 0 |
| 1108Kearns20 | Kearns | Aug-16 | 277 | No |  | 0-180 | 0 |
| 1108Kearns6 | Kearns | Aug-16 | 319 | Yes | 1 | 10-290 | 0 |
| 1608Vin1 | Vindula | Aug-16 | 173 | No |  | 0-15 | 0-1 |
| 1905MPRF11 | Mount Peter | May-16 | 229 | No |  | 0-265 | 0 |
| 1905MPRF12 | Mount Peter | May-16 | 229 | No |  | 0-306 | 0 |
| 1905MPRF15 | Mount Peter | May-16 | 229 | No |  | 0-160 | 0 |
| 1905MPRF6 | Mount Peter | May-16 | 361 | Yes | 3 | 0-402 | 0-3 |
| 1905MPS05 | Mount Peter | May-16 | 270 | No |  | 0-165 | 0-2 |
| 20170112Kearns20 | Kearns | Jan-17 | 221 | Yes | 1,2 | 0-337 | 0 |
| 20170112RPT3-S10 | Russet Park | Jan-17 | 285 | Yes | 3 | 0-185 | 0-4 |
| 20170112RPT3-S6 | Russet Park | Jan-17 | 243 | Yes | 1,2 | 0-370 | 0 |
| 20170112RPT4-S5 | Russet Park | Jan-17 | 320 | Yes | 2 | 5-402 | 0-1 |
| 20170112RPT4-S6 | Russet Park | Jan-17 | 165 | No |  | 15-180 | 0 |
| 20170112SC13 | Sandy Creek | Jan-17 | 249 | No |  | 0-566 | 0-9 |
| 20170112SC3 | Sandy Creek | Jan-17 | 242 | No |  | 5-446 | 0-7 |
| 20170112SC8 | Sandy Creek | Jan-17 | 165 | No |  | 0-686 | 0-7 |
| 20170215GC3 | Grey’s Creek | Feb-17 | 131 | Yes | 2 | 25-191 | 0 |
| 20170215Kearns13 | Kearns | Feb-17 | 230 | Yes | 1,2 | 105-425 | 0 |
| 20170215Kearns18 | Kearns | Feb-17 | 223 | Yes | 2 | 0-265 | 0 |
| 20170215RPT1-S1 | Russet Park | Feb-17 | 236 | No |  | 0-80 | 0 |
| 20170215RPT2-S5 | Russet Park | Feb-17 | 131 | No |  | 20-100 | 0 |
| 20170215RPT3-S8 | Russet Park | Feb-17 | 131 | No |  | 15-150 | 0 |
| 20170316FrankT1-6 | Frank's Creek | Mar-17 | 410 | No |  | 0-306 | 0-2 |
| 20170316FrankT2-1 | Frank's Creek | Mar-17 | 265 | No |  | 0-451 | 0-2 |
| 20170316GCT4-1 | Grey’s Creek | Mar-17 | 193 | Yes | 2 | 0-366 | 0 |
| 20170316GCT4-5 | Grey’s Creek | Mar-17 | 201 | No |  | 0-520 | 0 |
| 20170316GCT4-9 | Grey’s Creek | Mar-17 | 249 | Yes | 2 | 0-366 | 0-1 |
| 20170406FT1-1 | Frank's Creek | Apr-17 | 207 | Yes | 2 | 0-160 | 0 |
| 20170406FT2-7 | Frank's Creek | Apr-17 | 363 | No |  | 0-190 | 0-2 |
| 20170406WT2-10 | Whereat | Jun-17 | 246 | No |  | 0-10 | 0-6 |
| 20170410GC1 | Grey’s Creek | Apr-17 | 177 | No |  | 0-320 | 0 |
| 20170410GCT2-3 | Grey’s Creek | Apr-17 | 211 | Yes | 2 | 0-351 | 0 |
| 20170410Kearns13 | Kearns | Apr-17 | 261 | No |  | 0-311 | 0-2 |
| 20170410Kearns9 | Kearns | Apr-17 | 231 | No |  | 5-110 | 0 |
| 20170410VIT1-4 | Vindula | Apr-17 | 262 | Yes | 2 | 0-56 | 0 |
| 20170410VIT2-5 | Vindula | Apr-17 | 242 | Yes | 2 | 0-240 | 0 |
| 20170412FR14 | Frank’s Rainforest | Apr-17 | 175 | No |  | 0-180 | 0 |
| 20170412FR17&19 | Frank’s Rainforest | Apr-17 | 238 | Yes | 2 | 0-66 | 0 |
| 20170412SC14 | Sandy Creek | Apr-17 | 259 | No |  | 0-290 | 0-7 |
| 20170418RPT1-S10 | Russet Park | Apr-17 | 272 | No |  | 0-420 | 0-1 |
| 20170418RPT1-S8 | Russet Park | Apr-17 | 202 | No |  | 0-175 | 0 |
| 20170418RPT2-S7 | Russet Park | Apr-17 | 232 | No |  | 5-441 | 0-1 |
| 20170418RPT4-S10 | Russet Park | Apr-17 | 209 | No |  | 0-221 | 0-1 |
| 20170418RPT5-S8 | Russet Park | Apr-17 | 209 | No |  | 0-290 | 0-3 |
| 20170421Hussey2 | Hussey | Apr-17 | 318 | No |  | 0-366 | 0-3 |
| 20170503Hussey1 | Hussey | Mar-17 | 239 | Yes | 2 | 0-366 | 0 |
| 20170503RPT2-S8 | Russet Park | May-17 | 223 | Yes | 1 | 15-85 | 0 |
| 20170505VIT1-10 | Vindula | May-17 | 222 | No |  | 0-180 | 0 |
| 20170508Fal1 | Falcon | May-17 | 301 | No |  | 0-680 | 0-2 |
| 20170508SC1 | Sandy Creek | May-17 | 203 | Yes | 1 | 0-306 | 0 |
| 20170508SC7 | Sandy Creek | May-17 | 219 | No |  | 0-280 | 0 |
| 20170524Fal1 | Falcon | May-17 | 68 | No |  | 0-316 | 0 |
| 20170530GC1 | Grey’s Creek | May-17 | 140 | Yes | 2 | 0-85 | 0-3 |
| 20170530GC2 | Grey’s Creek | May-17 | 133 | No |  | 0-80 | 0-3 |
| 20170530GC3 | Grey’s Creek | May-17 | 147 | No |  | 0-75 | 0-3 |
| 20170530GC4 | Grey’s Creek | May-17 | 105 | Yes | 2 | 5-85 | 1-3 |
| 20170621FT1-2 | Frank's Creek | Jun-17 | 201 | No |  | 0-46 | 0-1 |
| 20170621GCT2A-1 | Grey’s Creek | Jun-17 | 124 | No |  | 0-10 | 0-2 |
| 20170622RPT4-S1,3 | Russet Park | Jun-17 | 228 | No |  | 15-90 | 0 |
| 20170717DCT2-7 | Draper Creek | Jul-17 | 147 | No |  | 20-170 | 0 |
| 20170717Vin1T1-4 | Vindula | Jul-17 | 56 | No |  | 5-5 | 1-3 |
| 20170803Harold8 | Harold | Aug-16 | 461 | No |  | 0-311 | 0-8 |
| 20170815DCT1-4&6 | Draper Creek | Aug-17 | 118 | No |  | 15-170 | 0 |
| 20170815DCT2-1&7 | Draper Creek | Aug-17 | 161 | No |  | 20-80 | 0 |
| 20170921SC12 | Sandy Creek | Sep-17 | 202 | No |  | 0-175 | 0 |
| 20170921SC4 | Sandy Creek | Sep-17 | 124 | No |  | 15-90 | 0 |
| 20171016RPT2-S8 | Russet Park | Oct-17 | 140 | No |  | 0-110 | 0 |
| 20171025VIT1-8 | Vindula | Oct-17 | 33 | Yes | 2 | 270-306 | 22-22 |
| 20171101SW2 | Swallow | Nov-17 | 180 | Yes | 2 | 0-175 | 0 |
| 20171106SC14 | Sandy Creek | Nov-17 | 29 | No |  | 250-265 | 2-4 |
| 20171106SC5 | Sandy Creek | Nov-17 | 29 | No |  | 250-311 | 2-2 |
| 20180301VIT2-7 | Vindula | Mar-18 | 123 | No |  | 0-25 | 0 |
| 20180510ST1 | Sikh Temple | May-18 | 291 | Yes | 2 | 0-75 | 0 |
| 20180510ST3 | Sikh Temple | May-18 | 222 | No |  | 0-5 | 0 |
| 20180521ST5 | Sikh Temple | May-18 | 393 | No |  | 0-61 | 0-1 |
| 20180528ST10 | Sikh Temple | May-18 | 231 | No |  | 15-285 | 0-2 |
| 20180619KK1 | Wrights Creek1 | Jun-18 | 747 | Yes | 2 | 0-565 | 0-1 |
| 20180619KK2 | Wrights Creek1 | Jun-18 | 433 | No |  | 0-626 | 0-3 |
| 20180619KK3 | Wrights Creek1 | Jun-18 | 545 | No |  | 15-556 | 0-2 |
| 20180619ST11 | Sikh Temple | Jun-18 | 329 | No |  | 0-190 | 0 |
| 20181203MS1 | Mt Sheridan | Dec-18 | 352 | Yes | 2 | 180-446 | 2-3 |
| 20190123Fan6 | Fantin Creek | Jan-19 | 222 | Yes | 1,2 | 0-351 | 0 |
| 20190220Fan6 | Fantin Creek | Feb-19 | 194 | No |  | 0-356 | 0-1 |
| 20190220Fan7 | Fantin Creek | Feb-19 | 258 | Yes | 1 | 0-541 | 0 |
| 20190220Mill18&19 | Gordonvale Mill | Feb-19 | 221 | Yes | 1,2 | 0-405 | 0 |
| 20190220Mill20+ | Gordonvale Mill | Feb-19 | 207 | Yes | 2 | 0-376 | 0 |
| 20190220Mill3&4 | Gordonvale Mill | Feb-19 | 143 | Yes | 2 | 0-280 | 0 |
| 20190311Mill1&4 | Gordonvale Mill | Mar-19 | 113 | Yes | 2 | 290-595 | 0 |
| 20190311Mill11-17 | Gordonvale Mill | Mar-19 | 266 | No |  | 0-770 | 0-1 |
| 20190311Mill14-17 | Gordonvale Mill | Mar-19 | 113 | Yes | 2 | 295-566 | 0-1 |
| 20190311Mill18,19,21,23 | Gordonvale Mill | Mar-19 | 113 | Yes | 2 | 360-556 | 0 |
| 20190311Mill20-22 | Gordonvale Mill | Mar-19 | 259 | Yes | 2 | 5-686 | 1-1 |
| 20190311Mill22,24,25 | Gordonvale Mill | Mar-19 | 113 | Yes | 2 | 290-595 | 0 |
| 20190311Mill24-28 | Gordonvale Mill | Mar-19 | 254 | Yes | 2 | 0-535 | 1-1 |
| 20190311Mill33-38 | Gordonvale Mill | Mar-19 | 231 | No |  | 0-456 | 0-1 |
| 20190311Mill40-50 | Gordonvale Mill | Mar-19 | 254 | No |  | 110-595 | 1-1 |
| 20190311Mill8&10 | Gordonvale Mill | Mar-19 | 259 | Yes | 2 | 0-840 | 1-1 |
| 20190328Fan2 | Fantin Creek | Mar-19 | 237 | Yes | 2 | 141-536 | 2-3 |
| 20190328Fan6 | Fantin Creek | Mar-19 | 237 | No |  | 5-420 | 1-1 |
| 20190328GF16 | Green Forest Road | Mar-19 | 237 | No |  | 265-451 | 1-1 |
| 20190328GF18 | Green Forest Road | Mar-19 | 237 | No |  | 80-441 | 1-1 |
| 20190328GF19 | Green Forest Road | Mar-19 | 237 | No |  | 255-345 | 4-6 |
| 20190328GF21 | Green Forest Road | Mar-19 | 222 | No |  | 0-290 | 0 |
| 20190328GF22 | Green Forest Road | Mar-19 | 237 | Yes | 2 | 85-435 | 1-1 |
| 20190328GF23 | Green Forest Road | Mar-19 | 237 | No |  | 160-586 | 2-3 |
| 20190328GF25 | Green Forest Road | Mar-19 | 237 | No |  | 260-515 | 4-4 |
| 20190328GF27 | Green Forest Road | Mar-19 | 237 | No |  | 170-355 | 1-1 |
| 20190328GF3 | Green Forest Road | Mar-19 | 237 | No |  | 260-595 | 2-2 |
| 20190328GF30 | Green Forest Road | Mar-19 | 237 | No |  | 265-536 | 3-4 |
| 20190328GF33 | Green Forest Road | Mar-19 | 237 | No |  | 255-490 | 6-9 |
| 20190328GF35 | Green Forest Road | Mar-19 | 237 | No |  | 260-415 | 3-4 |
| 20190328GF5 | Green Forest Road | Mar-19 | 237 | No |  | 170-580 | 2-4 |
| 20190328GF9,12,14 | Green Forest Road | Mar-19 | 237 | Yes | 1 | 275-556 | 0 |
| 20190430Fan6&10 | Fantin Creek | Apr-19 | 204 | No |  | 5-556 | 0 |
| 20190508Mill15,21,25 | Gordonvale Mill | May-19 | 167 | No |  | 0-296 | 0-1 |
| 20190529Fan6 | Fantin Creek | May-19 | 175 | No |  | 30-85 | 0 |
| 20190529GF10 | Green Forest Road | May-19 | 96 | No |  | 0-20 | 0 |
| 20190529GF11 | Green Forest Road | May-19 | 201 | No |  | 20-265 | 2-2 |
| 20190529GF13 | Green Forest Road | May-19 | 201 | No |  | 5-85 | 1-1 |
| 20190529GF21 | Green Forest Road | May-19 | 96 | No |  | 0-15 | 0 |
| 20190529GF23 | Green Forest Road | May-19 | 96 | No |  | 0-15 | 0 |
| 20190529GF24 | Green Forest Road | May-19 | 47 | No |  | 0-5 | 0 |
| 20190529GF25 | Green Forest Road | May-19 | 201 | No |  | 5-150 | 1-1 |
| 20190529GF26 | Green Forest Road | May-19 | 96 | No |  | 0-10 | 0 |
| 20190529GF27 | Green Forest Road | May-19 | 96 | No |  | 0 | 2 |
| 20190529GF29 | Green Forest Road | May-19 | 96 | No |  | 0-10 | 0-1 |
| 20190529GF30 | Green Forest Road | May-19 | 201 | No |  | 46-265 | 1-2 |
| 20190529GF31 | Green Forest Road | May-19 | 201 | No |  | 80-291 | 1-1 |
| 20190529GF32 | Green Forest Road | May-19 | 201 | No |  | 80-405 | 1-1 |
| 20190529GF33 | Green Forest Road | May-19 | 201 | No |  | 5-46 | 0 |
| 20190529GF34 | Green Forest Road | May-19 | 201 | No |  | 5-280 | 1-2 |
| 20190529GF35 | Green Forest Road | May-19 | 201 | No |  | 41-280 | 2-3 |
| 20190529GF39 | Green Forest Road | May-19 | 96 | No |  | 0-5 | 0 |
| 20190529GF40 | Green Forest Road | May-19 | 96 | No |  | 0-10 | 0 |
| 20190529GF47 | Green Forest Road | May-19 | 96 | No |  | 0-25 | 0 |
| 20190529GF48 | Green Forest Road | May-19 | 201 | No |  | 10-170 | 3-4 |
| 20190529GF49 | Green Forest Road | May-19 | 201 | No |  | 5-20 | 0 |
| 20190529GF50 | Green Forest Road | May-19 | 110 | No |  | 0-20 | 0-1 |
| 20190529GF8 | Green Forest Road | May-19 | 201 | No |  | 41-280 | 1-3 |
| 20190529GF9 | Green Forest Road | May-19 | 96 | No |  | 0-25 | 0 |
| 20190619Fan6 | Fantin Creek | Jun-19 | 154 | No |  | 90-290 | 0 |
| 20190620GF17 | Green Forest Road | Jun-19 | 179 | No |  | 160-340 | 1-1 |
| 20190620GF25 | Green Forest Road | Jun-19 | 179 | No |  | 5-270 | 0 |
| 20190620GF27 | Green Forest Road | Jun-19 | 74 | No |  | 0-5 | 0 |
| 20190620GF29 | Green Forest Road | Jun-19 | 179 | No |  | 150-301 | 2-4 |
| 20190620GF30 | Green Forest Road | Jun-19 | 179 | No |  | 206-311 | 11-12 |
| 20190620GF32 | Green Forest Road | Jun-19 | 179 | No |  | 5-20 | 0 |
| 2109Kearns20 | Kearns | Sep-16 | 173 | No |  | 5-275 | 0-2 |
| 2109Kearns4 | Kearns | Sep-16 | 278 | No |  | 15-175 | 0 |
| 2109RPT1-S7 | Russet Park | Sep-16 | 279 | No |  | 10-405 | 0-2 |
| 2109RPT1-S9 | Russet Park | Sep-16 | 342 | No |  | 0-252 | 0-3 |
| 2109Sandy9 | Sandy Creek | Sep-16 | 412 | No |  | 0-301 | 0-7 |
| 2109SC8 | Sandy Creek | Sep-16 | 363 | No |  | 0-311 | 0-9 |
| 2110Kearns3 | Kearns | Oct-16 | 248 | Yes | 2 | 5-180 | 0 |
| 2110RPT1-S8 | Russet Park | Oct-16 | 283 | No |  | 0-311 | 0-2 |
| 2110RPT2-S1 | Russet Park | Oct-16 | 311 | No |  | 0-211 | 0-4 |
| 2110RPT2-S4 | Russet Park | Oct-16 | 424 | No |  | 0-211 | 0-6 |
| 2110RPT2-S7 | Russet Park | Oct-16 | 298 | No |  | 0-221 | 0-1 |
| 2110RPT4-S10 | Russet Park | Oct-16 | 333 | No |  | 5-315 | 0 |
| 2110SC12 | Sandy Creek | Oct-16 | 283 | No |  | 0-211 | 0-9 |
| 2110SC15 | Sandy Creek | Oct-16 | 539 | No |  | 5-310 | 0-38 |
| 2110SC16 | Sandy Creek | Oct-16 | 304 | No |  | 0-360 | 0-17 |
| 2110SC20 | Sandy Creek | Oct-16 | 290 | No |  | 0-360 | 2-18 |
| 2206RPPad1 | Russet Park | Jun-16 | 166 | No |  | 0-51 | 0-2 |
| 2206RPPad3 | Russet Park | Jun-16 | 243 | No |  | 0-51 | 0-2 |
| 2308Rankin's | Grey’s Creek | Aug-16 | 97 | No |  | 0-10 | 0-2 |
| 2405RPRob's | Russet Park | May-16 | 286 | No |  | 0-260 | 0 |
