# Supplementary material for: Origin, behaviour, and genetics of reproductive workers in an invasive ant
Source: Front Zool. 2021 Mar 22;18:13. doi: 10.1186/s12983-021-00392-2 (PMC7986258; doi:10.1186/s12983-021-00392-2)
Supplement: Supplementary file 2 — Additional file 2 : Figure S1. Boxplot showing the proportion of normal workers in the queen transfer experiment by colony status and number of days since the start of the experiment until day 120. Figure S2. Forewing of a male A. gracilipes showing which veins were used to measure wing width and wing length Appendix S1. Additional information on the genotyping protocol including Table S5 which summarises the characteristic of the microsatellite loci. [file 12983_2021_392_MOESM2_ESM.docx]

**Additional file 2**

*


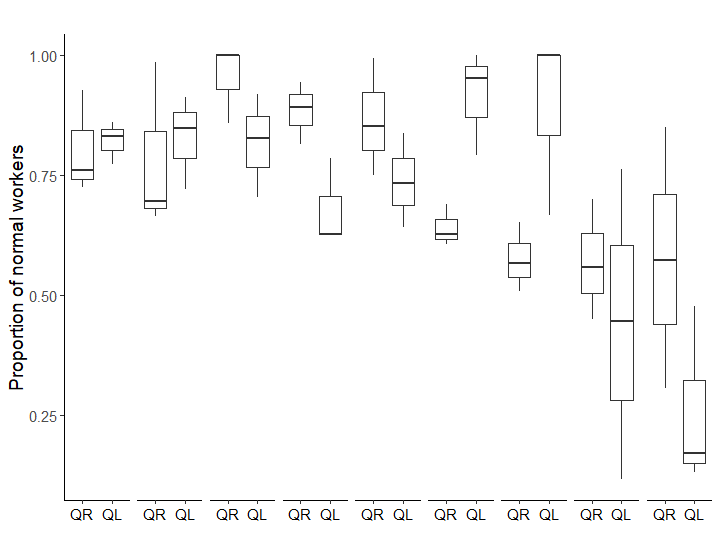


**

**

**

**⇩**

0 day

15 days

30 days

45 days

60 days

90 days

105 days

120 days

75 days

QR QL

QR QL

QR QL

QR QL

QR QL

QLm QRm

QLm QRm

QLm QRm

QLm QRm

**Figure S1.** Proportion of normal workers in the queen transfer experiment by colony status (QR=queenright, QL=queenless, N=3 for each) and number of days since the start of the experiment until day 120. **⇩** indicates when queens have been moved from the queenright subcolonies to the queenless subcolonies. After this date, queenright colonies became queenless (QLm) and queenless colonies became queenright (QRm). * indicates a significant difference between queenright and queenless colonies for the corresponding time (GLMM: binomial, Table 2, post hoc tests, *p<0.05, **p<0.01).


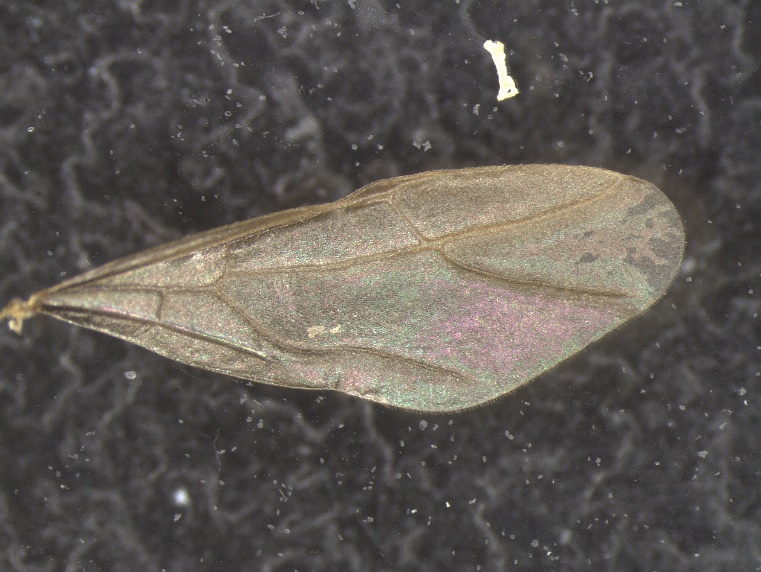


**Figure S2.** Forewing of a male *A. gracilipes* showing typical wing venation. Wing width (in blue) and wing length (in red) were always measured using the same wing veins as reference.

**Appendix S1: Genotyping protocol**

We washed each individual sample three times in distilled water to avoid contamination and extracted the DNA of whole ants using Puregene DNA isolation kits (Gentra Systems) with minor modifications to the supplier’s protocol (addition of 20µl of 200µg/ml Proteinase K and 2-hours water bath incubation after addition of the cell lysis solution). We resuspended the DNA pellets in 30μl DNA hydration solution and stored the DNA samples at 4°C. We used six microsatellite markers which have been found to be polymorphic for *A. gracilipes* (Table S5) . We conducted the polymerase chain reaction (PCR) amplification as in Drescher et al. (2007). We used a total reaction volume of 12.5μl with approximately 10ng of template DNA (2μl), 1 × PCR-buffer (1.25μl), 160μM dNTPs (1μl), 2.5μM of each primer (0.25μl) and 0.5U of Taq DNA polymerase (0.1μl, PEQLAB GmbH). The forward primers were labelled with fluorescent IR-700 or IR-800 dye (LI-COR). The cycle parameters were as follow: initial denaturation step at 3 min at 94°C, 30 cycles comprising 40 seconds of DNA denaturing at 94˚C, specific primer annealing temperature Table S5) for 40 seconds, 40 seconds DNA extension at 72˚C, and a final extension step of 3 minutes at 72˚C. We conducted a 1:10 dilution on the PCR products and analysed them on a LI-COR 4300 DNA Analyzer.

**Table S5.** Characteristics of microsatellite loci of *Anoplolepis gracilipes* used in this study. Ta: annealing temperature, bp: base pairs, N_I_ / N_C_: number of individuals successfulliy sequenced / number of colonies they were collected from

| Primer name | Sequence (5`- 3`) | Ta (°C) | Size range(bp)* | N alleles* | N_I_ / N_C_* | Publication |
| --- | --- | --- | --- | --- | --- | --- |
| Ano1 forward | ACTATCGTTTCTCTCACTAGG | 57 | 99 - 107 | 5 | 133/20 | Feldhaar et al. 2006 |
| Ano1 reverse | TCGGGTCTTAAAATACAGAGC |  |  |  |  |  |
| Ano3 forward | TCTTATCGTGCCTATTTGCTCG | 55 | 140 - 168 | 4 | 121/20 | Feldhaar et al. 2006 |
| Ano3 reverse | AGCGCAAACATTGCTAAGATG |  |  |  |  |  |
| Ano4 forward | TGACGTTTAGGCCTAATTTGACG | 60 | 156 - 174 | 3 | 129/21 | Feldhaar et al. 2006 |
| Ano4 reverse | TGAAGAGAGGATGAAAAAAGACG |  |  |  |  |  |
| Ano5 forward | ACGTGAATATTCGCTTGGACC | 60 | 116 - 122 | 4 | 74/18 | Feldhaar e tal. 2006 |
| Ano5 reverse | TCGGCTTCGCGTACTTCC |  |  |  |  |  |
| Ano6 forward | TGGTAGATTCCTTCTTATCG | 51 | 114 - 130 | 4 | 100/20 | Feldhaar et al. 2006 |
| Ano6 reverse | ACAAAATAAATACCCGCTGC |  |  |  |  |  |
| Ano9 forward | TCATGCTACCCTGAAGC | 52 | 149-155 | 3 | 124/20 | unpublished |
| Ano9 reverse | ACGTCTTATGGCAGTCG |  |  |  |  |  |

*calculated for present dataset

References

Drescher J, Blüthgen N, Feldhaar H. Population structure and intraspecific aggression in the invasive ant species *Anoplolepis gracilipes* in Malaysian Borneo. Mol Ecol. 2007;16:1453–65.

Feldhaar H, Drescher J, Blüthgen N. Characterization of microsatellite markers for the invasive ant species *Anoplolepis gracilipes*. Mol Ecol Notes. 2006;6:912–4.
